# Effects of the Autophagy-Inhibiting Agent Chloroquine on Acute Myeloid Leukemia Cells; Characterization of Patient Heterogeneity

**DOI:** 10.3390/jpm11080779

**Published:** 2021-08-10

**Authors:** Ida Sofie Grønningsæter, Håkon Reikvam, Elise Aasebø, Sushma Bartaula-Brevik, Maria Hernandez-Valladares, Frode Selheim, Frode S. Berven, Tor Henrik Tvedt, Øystein Bruserud, Kimberley Joanne Hatfield

**Affiliations:** 1 Department of Clinical Science, University of Bergen, N-5021 Bergen, Norway; ida.groenningsaeter@gmail.com (I.S.G.); hakon.reikvam@uib.no (H.R.); elise.aasebo@uib.no (E.A.); sbartaulabrevik@gmail.com (S.B.-B.); 2 Department of Medicine, Akershus University Hospital, N-1478 Lørenskog, Norway; 3 Department of Medicine, Haukeland University Hospital, N-5021 Bergen, Norway; tor.henrik.anderson.tvedt@helse-fonna.no; 4 The Proteomics Facility of the University of Bergen (PROBE), Department of Biomedicine, University of Bergen, N-5009 Bergen, Norway; Maria.Hernandez-Valladares@uib.no (M.H.-V.); Frode.Selheim@uib.no (F.S.); Frode.Berven@uib.no (F.S.B.); 5 The Department of Biomedicine, University of Bergen, N-5009 Bergen, Norway; 6 Department of Hematology, Oslo University Hospital—The National Hospital, N-0372 Oslo, Norway; 7 Department of Immunology and Transfusion Medicine, Haukeland University Hospital, N-5009 Bergen, Norway

**Keywords:** acute myeloid leukemia, apoptosis, chemokine, chloroquine, cytokine, proliferation, protein profiling

## Abstract

Autophagy is a highly conserved cellular degradation process that prevents cell damage and promotes cell survival, and clinical efforts have exploited autophagy inhibition as a therapeutic strategy in cancer. Chloroquine is a well-known antimalarial agent that inhibits late-stage autophagy. We evaluated the effects of chloroquine on cell viability and proliferation of acute myeloid leukemia acute myeloid leukemia (AML) cells derived from 81 AML patients. Our results show that chloroquine decreased AML cell viability and proliferation for the majority of patients. Furthermore, a subgroup of AML patients showed a greater susceptibility to chloroquine, and using hierarchical cluster analysis, we identified 99 genes upregulated in this patient subgroup, including several genes related to leukemogenesis. The combination of chloroquine with low-dose cytarabine had an additive inhibitory effect on AML cell proliferation. Finally, a minority of patients showed increased extracellular constitutive mediator release in the presence of chloroquine, which was associated with strong antiproliferative effects of chloroquine as well as cytarabine. We conclude that chloroquine has antileukemic activity and should be further explored as a therapeutic drug against AML in combination with other cytotoxic or metabolic drugs; however, due to the patient heterogeneity, chloroquine therapy will probably be effective only for selected patients.

## 1. Introduction

Acute myeloid leukemia (AML) is the most common type of acute leukemia in adults. It is an aggressive malignancy characterized by the accumulation of immature leukemic cells in the bone marrow [1,2]. Current treatment of AML relies largely on intensive chemotherapy, possibly followed by allogeneic hematopoietic stem cell transplantation (allo-HSCT), and intensive chemotherapy alone or combined with allotransplantation is the only curative treatment of the disease. However, the median age of AML patients at diagnosis is 65–70 years, and elderly patients >70–80 years have a dismal outcome because many elderly patients cannot tolerate the most intensive therapy, favorable genetic abnormalities are less frequent, and chemoresistant disease (e.g., AML secondary to cytotoxic drugs or previous hematological malignancies) is more frequent [1,2,3]. Long-term AML-free survival (i.e., overall and progression-free survival) is only 35–45% even for younger patients below 60–70 years of age without severe comorbidity who can tolerate the most intensive conventional therapy possibly including allogeneic stem cell transplantation [1]. Although advances in treatment have significantly improved outcomes for younger patients, there is still a strong need for new and more effective agents, or efficient combinations of agents, with a less toxic effect, to treat AML, also including those patients unable to tolerate intensive treatment [1,4].

AML is a very heterogeneous disease both with respect to karyotype as well as molecular genetic abnormalities [2,3]. A wide range of mutations have been detected in this disease, and some of these mutations (e.g., fms related receptor tyrosine kinase 3 (*FLT3*) and isocitrate dehydrogenase (*IDH*) mutations) are targets for new pharmacotherapy [1], and the B-cell lymphoma 2 (BCL-2) inhibitor venetoclax is another example of targeted therapy [1]. The risk of relapse after intensive and potentially curative conventional therapy varies between patients, and patients can be classified into various prognostic subsets (i.e., different risk of relapse) based on karyotypic and molecular genetic abnormalities [2,4]. Furthermore, patients who cannot receive the most intensive therapy should receive AML-stabilizing treatment, usually with hypomethylating agents possibly in combination with venetoclax [1,3]. The new targeted therapies can be tried either as a part of the conventional intensive therapy (e.g., midostaurin for patients with *FLT3* abnormalities) or as a part of disease-stabilizing treatment (e.g., venetoclax, IDH inhibitors) [1]. Targeting autophagy is another new therapeutic strategy being considered for treatment of cancer [5], including AML [6,7]. 

Autophagy is a cellular recycle mechanism where cells digest their own cytoplasmic components; damaged proteins and organelles are thereby eliminated, and cellular homeostasis is maintained. The autophagic process can be tumor suppressive through the removal of damaged organelles or oncogenic substrates [8,9], but it can also contribute to carcinogenesis by providing energy and maintaining metabolism for cancer cells under stressful conditions [10] and sustain growth and survival when cancer cells are challenged by cytotoxic therapies [11]. Given its essential role in cell growth and survival, autophagy is therefore being investigated as a target for therapeutic intervention.

Chloroquine is a clinically available drug that is shown to inhibit the last stage of autophagy. The drug was discovered in 1934 and initially used to treat malaria for which it has obtained approval from the U.S. Food and Drug Administration (FDA). These last decades, chloroquine and analogs have further been widely tested for their anticancer activity against a variety of cancer types [12]. The precise mechanism of the anticancer effects of chloroquine remains unclear; however, scientific reports suggest that the autophagy-specific mechanism of action of chloroquine is at least partially responsible, though other molecular mechanisms independent of autophagy may also contribute [13]. Chloroquine inhibits autophagy by raising lysosomal pH that leads to inhibition of the fusion of autophagosomes with lysosomes, disrupting the lysosomal protein degradation [14]. As chloroquine and its derivate hydroxychloroquine are FDA-approved drugs, they are therefore the main agents that have been tested in the clinic to treat cancer through inhibition of autophagy. There are multiple ongoing clinical trials with these drugs alone or in combination with other anticancer agents [5,15]. In addition, chloroquine has been shown to have an effect on the chemosensitization of cancer cells, independent of its autophagy-dependent anticancer effects [16,17].

Several recent reviews have emphasized that the effect of autophagy in human AML is context-dependent; autophagy can have a tumor-suppressive effect during early steps of leukemogenesis through degradation of oncogenic proteins, while a tumor-promoting effect has been described during proliferation of AML cells (i.e., disease development/progression), and autophagy can mediate prosurvival protective mechanisms during chemotherapy or be induced as a protective cellular response during exposure to antileukemic drugs [18,19]. Thus, the effect of autophagy in AML is context-dependent, and because AML is a highly heterogeneous disease, our hypothesis is that the effect of autophagy inhibition will vary between patients. Even though several clinical studies have investigated the possible use of chloroquine in cancer treatment including AML therapy [13,18,19,20,21,22,23,24,25,26], to the best of our knowledge, none of the previous AML studies have characterized the patient heterogeneity with regard to susceptibility to chloroquine. Although several chloroquine analogs have been developed, studies of chloroquine are still relevant as this drug may serve as a model drug for this class of anticancer agents [24]. In the present study, we therefore aimed to evaluate the antileukemic effects of chloroquine, in particular effects on cell proliferation, survival, and constitutive soluble mediator release by primary AML cells derived from a large group of consecutive and thereby unselected AML patients. Finally, we examined the antileukemic effects of chloroquine in combination with cytarabine/cytosine arabinoside (AraC), and investigated whether we could identify a subset among the heterogeneous AML patients that might benefit from chloroquine treatment.

## 2. Materials and Methods

### 2.1. Preparation of Primary AML Cells 

The study was conducted after approval by the Regional Ethics Committee (REK) III 060.02, (100602), REK Vest 2013-634 (190313), REK Vest 2015/1410 (190615), and samples collected after written informed consent from all patients. The characteristics of AML patients included in the study are shown in Table S1. Patients with the FAB M3 subtype of AML were excluded. Peripheral blood mononuclear cells were isolated from patients with at least 80% AML cells among circulating leukocytes. Leukemia cell preparation was performed using density gradient separation (density 1.077 g/mL; Lymphoprep, Serumwerk Bernburg AG for Alere Technologies AS, Oslo, Norway), resulting in cell populations with >90% leukemia cells for all patients, where contaminating cells were mainly small lymphocytes [27,28,29]. The isolated AML blasts were immediately cryopreserved in RPMI 1640 medium (Sigma-Aldrich, St. Louis, MO, USA) with 10% dimethylsulfoxide and 20% inactivated fetal bovine serum (Biowest, Riverside, MO, USA). Cells were stored in liquid nitrogen until the cryopreserved cells were thawed, counted, and used directly in the experimental studies.

### 2.2. Normal Cells

Umbilical cord blood (UCB) cells were obtained from five donors after written informed consent (REK Vest 2015/1759 (051115), 2017/305 (070417)). Mononuclear cells were enriched in a similar manner to AML cell populations, by using density gradient separation, and stored in liquid nitrogen until used in experiments.

Cryopreserved human primary mesenchymal stem cells (MSCs) from the bone marrow of a healthy donor (MSC24539, 24-year old female Caucasian) were purchased from Lonza in passage two (Cambrex BioScience, Walkersville, MD, USA) and were expanded in complete MSC growth medium (MSCGM™; Lonza) with 10% inactivated fetal bovine serum (Biowest, Riverside, MO, USA) and 4 mM L-glutamine (Sigma-Aldrich, St. Louis, MO, USA). 

### 2.3. AML Cell Culture Medium 

Serum-free Stem Span SFEM™ medium (Stem Cell Technologies, Vancouver, BC, Canada) supplemented with exogenous granulocyte-colony stimulating factor (G-CSF), stem cell factor (SCF), and fms-related tyrosine kinase 3 ligand (FLT3-L) was used in all cell culture experiments with patient AML cells except coculture studies with MSCs (described below). Growth factors were purchased from Peprotech (Rocky Hill, NJ, USA) and used at a final concentration of 20 ng/mL. This medium has been designed for the culture of normal hematopoietic stem and progenitor cells, and it was supplemented with these three growth factors for the culture of normal immature myeloid cells. Mononuclear umbilical cord blood (UCB) cells include lymphoid cells, but also immature myeloid cells (e.g., umbilical cord stem cells); control experiments showed that normal mature lymphoid cells show very low or no proliferation when incubated in this medium. We therefore regard the UCB cell population to include mainly proliferating immature myeloid progenitor and stem cells. 

AML cell lines were cultured in RPMI-1640 medium supplemented with streptomycin-penicillin (50 µg/mL), 2 mM L-glutamine, and 10% heat-inactivated fetal bovine serum.

### 2.4. Reagents

Chloroquine and bafilomycin A1 were purchased from Sigma-Aldrich (St. Louis, MO, USA, cat. no. C6628 and 196000, respectively), and cytarabine (AraC) was obtained from Pfizer (New York City, NY, USA). All drugs were prepared according to datasheets provided by the distributors. Stock solutions of chloroquine were diluted in phosphate-buffered saline (PBS), sterile-filtered (0.22 µm), and stored in small aliquots at −20 °C until used. Aliquots were thawed only once and diluted with their respective solvents to obtain the desired final concentrations.

### 2.5. In Vitro Cell Culture Studies 

Suspension cultures of AML cells alone were seeded in triplicates (1 × 10^6^ /mL, 200 μL medium/well) in flat-bottomed 96-well microtiter plates (Nucleon™; Nunc, Roskilde, Denmark), and cultures were then incubated with or without drugs in StemSpan medium supplemented with exogenous cytokines. AML cell proliferation was investigated using a 3H-thymidine incorporation assay [30]. After 6 days of incubation at 37 °C in a humidified atmosphere of 5% CO_2_, 20 μL of 37 kBq 3H-thymidine (TRA 310, Amersham, UK) in saline was added per well, and nuclear incorporation was determined 22 h later. For each drug concentration, the effect on proliferation was calculated by comparing cell proliferation (cpm values) as the percentage of untreated cultures. The median of triplicate cultures was used for all calculations, and detectable incorporation was defined as >1000 counts per minute (cpm).

For cocultures of AML cells and primary mesenchymal stem cells (MSCs), the MSCs were trypsinated and used in cocultures in passage four. Cocultures were prepared as described previously [31], by adding MSCs to the lower chamber and AML cells to the upper chamber of transwell plates (Costar 3401; 0.4 µm pore size, Costar, Cambridge, MA, USA) in complete MSC medium, thus allowing no direct MSC-AML cell contact. Cocultures were incubated for 2 days with or without 5 µM chloroquine before 280 kBq/well 3H-thymidine was added, and proliferation of both MSCs and AML cells after 3-day coculture was determined as described in detail previously [32]. For cocultures, triplicates derived from the same transwell culture were used for all calculations.

Soluble mediator levels were analyzed in both single culture and coculture supernatants. AML cells were cultured for 48 h in cytokine-supplemented Stem Span SFEM™ medium (1 × 10^6^ /mL, 1 mL per well) in 24-well culture plates (Nucleon™; Nunc) with or without 5 µM chloroquine. All supernatants were collected and stored at −80 °C before analysis. In addition, cell culture supernatants were harvested from MSC-AML cocultures (prepared in MSC medium) with or without 5 µM chloroquine for two days. Subsequently, 19 mediators were analyzed by Luminex^®^ bead-based multiplex assays strictly according to the distributors’ protocol (LXSAHM-17, R&D Systems; Minnesota, MN, USA).

The CytoID^®^ autophagy detection kit (ENZO, Life Sciences, Farmingdale, NY, USA) was used to measure autophagic vesicles (pre-autophagosomes, autophagosomes, and auto(phago)lysosomes) in two human AML cell lines, HL60 and MOLM-13 (obtained from the American Type Culture Collection (ATCC); cell identity confirmed for both cell lines). Cell lines were cultured for 18 h (10^6^ cells/mL) in 24-well culture plates (Nucleon™, Nunc) with or without chloroquine (2.5–60 µM) before analysis using the CytoID detection kit strictly according to the manufacturer’s instructions. The BD FACSVerse flow cytometer (BD Biosciences) was used to collect 10,000 events for each sample.

### 2.6. RNA Preparation, Labeling, and Microarray Hybridization

All microarray experiments were performed using the Illumina iScan Reader, which is based upon fluorescence detection of biotin-labeled cRNA. 300 ng of total RNA from each sample was reversely transcribed, amplified, and Biotin-16-UTP–labeled, using the Illumina^®^ TotalPrep™ RNA amplification kit (Applied Biosystems/Ambion, Foster City, CA, USA). Amount and quality of the biotin-labeled cRNA were controlled by both NanoDrop^®^ spectrophotometer (NanoDrop Technologies, Inc. Wilmington, DE, USA), and Agilent 2100 Bioanalyzer (Agilent Technologies, Inc., Palo Alto, CA, USA). 750 ng of biotin-labeled cRNA was hybridized to the HumanHT-12 V4 Expression BeadChip according to manufacturer’s instructions. The HumanHT-12 V4 BeadChip targets 47,231 probes derived primarily from genes in the NCBI RefSeq database (Release 38). The data from the scanning of arrays on Illumina iScan Reader was investigated in GenomeStudio (Illumina Inc., Hayward, CA, USA) and J-Express 2012 (MolMine AS, Bergen, Norway) for quality control measures [33]. Before being compiled into an expression profile data matrix, all arrays within each experiment were quantile normalized to be comparable. We used the analysis of variance (ANOVA), and by setting an F-score > 1.0 and a fold change (FC) value > 1.0, we identified genes differently expressed between the two patient populations. The genes encoding proteins with a known function were classified using the PANTHER (protein annotation through evolutionary relationship) classification system (version 14.0) [34].

### 2.7. Flow Cytometric Analyses of Cell Viability 

The percentage of viable, apoptotic, and necrotic primary AML cells were determined by flow cytometry using the ApoptestTM–FITC kit (NeXins Research, Kattendijke, the Netherlands) in accordance with the manufacturer’s instructions as previously described [35]. Cells were seeded into wells (1 × 10^6^/mL) and added either 2.5 or 5 µM chloroquine and/or 0.0125 µM cytarabine, while cells cultured in medium alone were used as controls. After 48 h of incubation at 37 °C in a humidified atmosphere of 5% CO_2_, cells were analyzed using a BD FACSVerse flow cytometer (BD Biosciences; Franklin Lakes, NJ, USA). Doublets were excluded by gating forward scatter (FSC)-height and FSC-area, and side scatter (SSC)-height and SSC-area. 10,000 events were collected for each sample. 

### 2.8. Mutational Analyses

Submicroscopic mutation profiling of 54 genes frequently mutated in myeloid leukemias was performed using the Illumina’s TruSight Myeloid Gene Panel as described in detail previously [36].

### 2.9. Proteomic Analyses of Primary Human AML Cells

Our methods for the preparation of AML cell samples and the methods for proteomic sample preparation and LC-MS/MS analysis have been described in detail previously [37]. The 16 LC-MS/MS raw files analyzed in the present study are a subset of the 41 raw files previously used in this publication, deposited to the ProteomeXchange consortium via the PRIDE partner repository with dataset identifier PXD014997. In the current study, we have re-analyzed these 16 LC-MS/MS raw files also used in the previous publication [37]. The 16 raw files were searched in MaxQuant (version 1.6.17.0) against the concatenated forward and reversed-decoy Swiss-Prot Homo sapiens database version downloaded 10 May 2021 [38,39,40]. MaxQuant parameters and statistical analyses were performed as described in the previous publication [37].

### 2.10. Statistical and Bioinformatical Analyses

Mann–Whitney U, Wilcoxon signed rank test and Kruskal–Wallis H-test with Dunn’s post hoc test, and Fisher’s exact test were used for statistical comparisons of AML patient cells. ANOVA, using F-score > 1.0 and a fold change (FC) value > 1.0, was used to identify genes in the gene expression analysis. Analysis and graphical presentations were done using IBM Statistical Package for the Social Sciences^®^ (SPSS^®^) v.23.0 (IBM SPSS statistics Inc., Chicago, IL, USA) and GraphPad^®^ prism version software v.5.02 (Graph Pad Software, Inc., San Diego, CA, USA). The flow cytometry data was analyzed using FlowJo™ v.10.3 software (Tree Star, Inc., Ashland, OR, USA). Differences were regarded as significant when *p* < 0.05.

## 3. Results

### 3.1. The Patient Population

As described in the material and methods section, we included 81 consecutive patients in our present study; all these patients were included during a defined time period and from a defined geographical area. The overall characteristics of the patients are summarized in Table S1. The median age of the patients was 67.5 years, and the majority of patients were de novo AML, while 14 patients had AML secondary to myelodysplastic syndrome/chronic myeloproliferative disease. A normal karyotype was seen for nearly half of the patients, whereas favorable karyotypes were observed for only a small minority of patients and nearly one-third of the patients had *FLT3*-ITD, and 28 out of 73 patients had nucleophosmin 1 (*NPM1*) abnormalities. All these characteristics are as expected when investigating a consecutive group of patients including a large subset of elderly patients above 70 years of age [1,2,3,4]. Only 39 patients received intensive and potentially curative treatment, and only 15 of these patients had long-term AML-free survival (all patients observed for at least 3 years).

### 3.2. Initial In Vitro Screening of the Antiproliferative Effects of Chloroquine on AML Primary Cells and Mononuclear Umbilical Cord-Derived Cells

**Figure 1:**
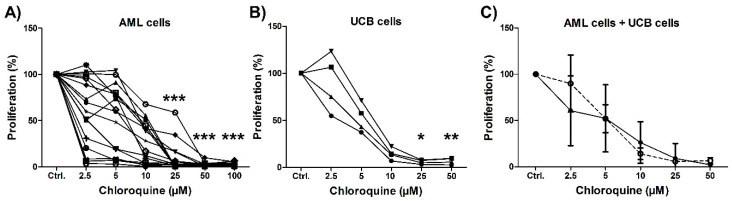
Initial in vitro drug screening study of chloroquine on patient-derived AML cells and UCB mononuclear cells. Cells were treated with chloroquine for six days before 3H-thymidine was added to cultures for an additional 22 h of incubation, before proliferation (nuclear incorporation) was determined by liquid scintillation counting. (**A**) Acute myeloid leukemia (AML) cells derived from 17 patients were treated with chloroquine at six different concentrations (2.5, 5, 10, 25, 50, and 100 µM), and (**B**) Umbilical cord blood (UCB) mononuclear cells from four donors were treated with chloroquine at five different concentrations (2.5, 5, 10, 25, and 50 µM). Detectable incorporation was defined as >1000 counts per minute (cpm). Results are shown as the percent proliferation of chloroquine-treated cultures compared to their respective untreated control cultures (set to 100%). At lower concentrations (2.5 and 5 µM) there was a varied sensitivity towards chloroquine, but at higher concentrations (10–100 µM) all samples showed decreased proliferation compared to untreated controls, * = *p*-value < 0.05, ** = *p*-value < 0.01, *** = *p*-value < 0.0001, Kruskal–Wallis, Dunn’s post-hoc test. (**C**) The figure shows the overall mean cell proliferation with SD, for all 17 AML patients (solid line) and four UCB donors (stippled line). There were no significant differences between the anti-proliferative effects of chloroquine on AML compared to UCB cells.

Initial studies to evaluate the effects of various concentrations of the drug chloroquine (2.5–100 µM) were conducted using the 3H-thymidine assay for AML cells derived from 17 patients Chloroquine had dose-dependent antiproliferative effects both for primary AML cells, although the sensitivity toward drugs varied considerably between AML patients at the lowest concentrations of 2.5 and 5 µM (Figure 1). None of the AML patient cells showed any proliferation after treatment with 50 µM chloroquine or higher concentrations (Figure 1). The aim of our study was to characterize patient heterogeneity, and we therefore chose to use chloroquine concentrations of 2.5 and 5 µM in the following experiments. Cytarabine concentrations were also based on dose-response experiments and we selected relatively low levels that allowed detection of differences between patients (data not shown).

We also investigated the effects of chloroquine on umbilical cord blood-derived cells (Figure 1). Chloroquine showed a dose-dependent antiproliferative effect on these normal cells that was similar to the effect on AML cells. This observation shows that chloroquine has an antiproliferative effect also toward normal cells, probably also for the normal myeloid stem/progenitor cells found in the mononuclear UCB cell populations. 

### 3.3. AML Cell Proliferation Is Inhibited by Chloroquine Alone and in Combination with Cytarabine

**Figure 2:**
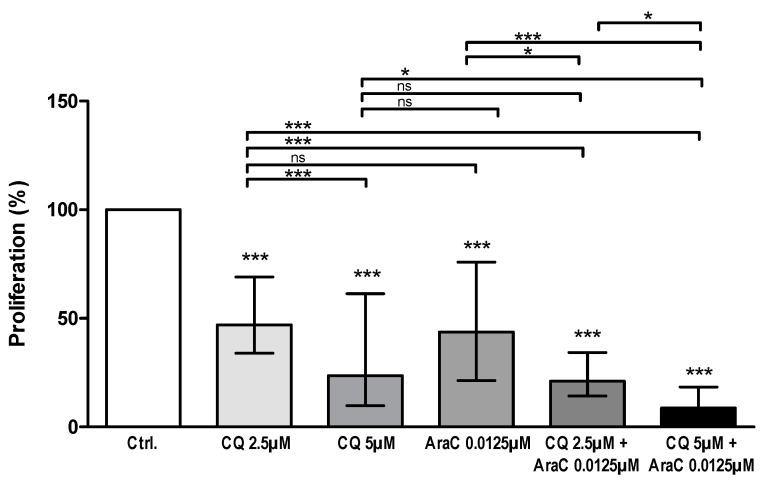
Effect of chloroquine (CQ) alone or in combination with cytarabine (AraC) on cytokine-dependent AML cell proliferation. AML cells from 81 consecutive patients were cultured for seven days in medium alone (control cultures) or treated with CQ (2.5 and 5 µM), cytarabine/AraC (0.0125 µM) or CQ in combination with cytarabine/AraC. The 3H–thymidine assay was used to measure cell proliferation. Detectable proliferation defined as >1000 cpm in untreated control cultures, was observed for 69 patients. Proliferation is shown as the median levels (with 25–75% percentiles), and proliferation in treated cultures is shown as percent proliferation of the drug-free controls (set to 100%). Significant effects were calculated using Mann–Whitney U-test for comparisons between drug-containing and drug-free controls (shown as asterisks above bars), and Kruskal–Wallis with Dunn’s post hoc test for comparison between the different groups (asterisks above brackets) (ns = not significant, * *p*-value = 0.05, *** *p*-value = 0.0001).

We investigated the effects of chloroquine alone (2.5 and 5 µM) on AML cell proliferation for 81 patients, using the 3H-thymidine incorporation assay. The two lowest concentrations (2.5 and 5 µM, see above) were chosen to characterize patient heterogeneity and also to be able to test chloroquine in combination with cytarabine. Detectable cell proliferation (>1000 cpm) was observed in drug-free controls (cells cultured in medium alone) for 69 patients, and further statistical analysis was therefore based on the results for these patients. When comparing the overall results, a highly significant antiproliferative effect of chloroquine was observed with both concentrations of chloroquine compared to untreated control cultures (*p*-value < 0.0001, Mann–Whitney U-test; Figure 2). As expected, cytarabine (0.0125 µM) also had a significant inhibitory effect on AML cell proliferation compared to untreated controls (Figure 2). However, the effect of cytarabine was not significantly different from any of the chloroquine concentrations tested alone when comparing overall effects on all patients (Kruskal–Wallis test, Dunn´s post hoc test; Figure 2). Furthermore, we combined chloroquine (2.5 and 5 µM) with cytarabine (0.0125 µM), and both concentrations of chloroquine in combination with cytarabine 0.0125 µM showed an additional inhibitory effect compared to chloroquine or cytarabine alone (*p*-value < 0.05, Kruskal–Wallis test, Dunn´s post hoc test; Figure 2).

Despite the statistical significances observed for the overall results (Figure 2), we emphasize there were exceptional patients in all the compared groups. The median and variation ranges illustrate this, i.e., proliferation in percent after drug treatment compared to the medium control. The effect of treatment with chloroquine 2.5 μM showed a wide variation between patients ranging from growth enhancement to a proliferation corresponding to only 3% of the drug-free control (median effect 47% of the control, range 3–183%); this variation is similar to the observations in our initial dose-response experiments (Figure 1). A wide variation toward drug treatment was also seen for chloroquine 5 μM (median 24%, range < 1–491%), AraC/cytarabine (median 43%, range 3–148%), chloroquine 2.5 μM plus AraC 0.0125 μM (median 21%, range < 1–147%) and chloroquine 5 μM plus AraC 0.0125 μM (median 9%, range < 1–128%). However, as can be seen from Figure 2, the exceptional patients with increased proliferation after drug treatment were relatively few.

The antiproliferative effect induced by chloroquine 2.5 μM among the various patients showed no significant association with differentiation (FAB classification, CD34 expression), karyotype, *NPM1* or *FLT3* mutations), and it did not differ between patients with secondary and de novo AML (data not shown). Finally, 33 patients completed intensive induction and consolidation treatment (some including allogeneic stem cell transplantation) according to the ELN guidelines [4], but the antiproliferative effect of chloroquine 2.5 μM did not differ between long-term AML-free survivors observed for at least 5 years and patients dying from leukemia relapse (data not shown). 

Additional mutational analyses were available for 15 patients; these patients were randomly selected from a consecutive group of patients admitted to our hospital for AML therapy (Table S2). All these 15 patients were unfit for intensive therapy, including hypomethylating agents. As expected, these patients had relatively high age (median age 73 years, range 48–78 years) and many of them had high-risk disease, e.g., secondary AML, complex karyotype, *TP53* mutations. Although these additional mutational data were available only for this small group of patients, the results illustrate that patients both with strong and weak antiproliferative effects of chloroquine 2.5 μM (i.e., less or more than 50% reduction of cytokine-dependent cell proliferation) are very heterogeneous with regard to AML-associated mutations.

Taken together, these overall results show that the antiproliferative effect of chloroquine 2.5 μM shows a considerable variation between patients, but this variation is not associated with any of the established biomarkers of high-risk AML (i.e., karyotype, *NPM1* or *FLT3* mutations) or with the survival of patients receiving potentially curative intensive therapy.

Additional experiments suggest that chloroquine inhibits/modulates autophagy in AML cells. First, we investigated whether chloroquine inhibits autophagy in the AML cell lines HL60 and MOLM-13 using the CytoID autophagy detection kit, and then a dose-dependent accumulation of autophagic compartments was observed with increasing amounts of chloroquine (Figure S1). Accumulation of autophagic compartments reached statistical significance for both cell lines when using chloroquine 60 μM and a more than four-fold increase was seen for MOLM-13 indicating a higher autophagy flux for this cell line compared to HL60 (Figure S1; *p* < 0.0001). These observations show that chloroquine modulates autophagy in AML cells, but the level of autophagy and the effect of chloroquine vary between AML cell lines. Second, the effect of bafilomycin A1 on AML cell proliferation was investigated for an unselected subset of 33 patients. Bafilomycin was tested at 1, 5, and 10 nM, and the highest bafilomycin concentration had an antiproliferative effect ranging from no inhibition to >90% inhibition. Both bafilomycin A1 10 nM and chloroquine 2.5 μM had a strong anti-proliferative effect (i.e., corresponding to >50% reduction compared with the corresponding medium controls) for 11 patients, both drugs showed a weaker inhibition for 12 patients and divergent effects (i.e., strong effect for only one of the two drugs) for 10 patients; this association between the effects of bafilomycin A1 and chloroquine reached statistical significance (Fisher’s exact test, *p* = 0.033; data not shown). 

### 3.4. Proteomic Comparison of Primary AML Cells Derived from Patients with High and Low Susceptibility to Chloroquine

Our present study included primary cells derived at the first time of diagnosis for 30 patients that completed AML treatment with intensive induction therapy, 2 or 3 consolidation cycles, and possibly allogeneic stem cell transplantation as a final consolidation treatment [1,4]. All these patients were below 65 years of age. Our proteomic studies were based on a consecutive subset of 19 (out of the 30) patients who completed the intensive treatment; two of these patients did not show cytokine-dependent proliferation. We investigated the proteomic AML cell profiles for eight patients where treatment with chloroquine 2.5 μM had an antiproliferative effect less than 40% compared to the proliferation of drug-free control cultures, the profiles for these eight patients were compared with eight other patients where chloroquine showed a stronger antiproliferative effect corresponding to at least 50% inhibition compared with the controls. One of the 17 patients with detectable cytokine-dependent proliferation showed an intermediate antiproliferative effect corresponding to 46% inhibition and was excluded from the proteomic comparison. Thus, we compared the proteomic profiles for two contrasting groups that included eight patients each.

A total of 5476 proteins could be quantified and only 55 of them differed significantly when comparing AML cells with strong and weak antiproliferative effects of chloroquine. However, autophagy is a complex multistep process involving organellar trafficking and fusion, and several of the differing proteins important for regulation of autophagy are localized to lysosomes or endosomes/endoplasmic reticulum, or they are involved in mitophagy/mitochondrial metabolism (Table 1). A subset of proteins is important for intracellular signaling or transcriptional regulation, whereas relatively few of the proteins are extracellular proteins or cell surface proteins/adhesion molecules. Thus, several of the proteins showing significantly different levels are important for the regulation of autophagy, but the levels of proteins included in autophagy-associated molecular complexes (i.e., the ULK1, PI3K, Atg9, and Atg12 conjugation complexes; see the Autophagy Database www.tanpaku.org/autophagy/index.html, accessed on 20 May 2021) did not differ between the two groups. Taken together, these results suggest that differences in the susceptibility to the antiproliferative effect of chloroquine depend on differences in the regulation of autophagy.

### 3.5. Chloroquine Inhibits AML Cell Proliferation in Cocultures with MSCs

**Figure 3:**
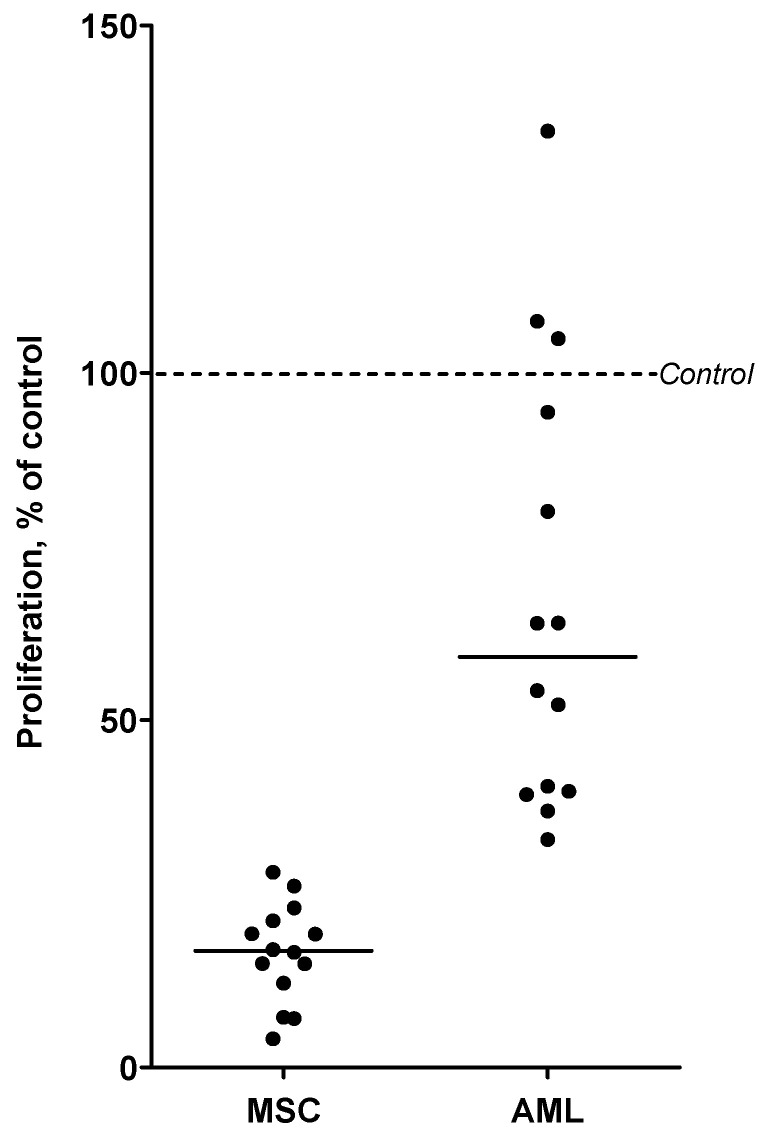
The effect of chloroquine (CQ) on the proliferation of primary AML cells and normal MSCs grown in coculture. The two cell populations were separated by a semipermeable membrane. Cocultures of AML cells (14 patients tested) and normal MSCs derived from a healthy donor were treated with 5 µM chloroquine for three days. Proliferation was measured using the 3H-thymidine incorporation assay for both AML cells and MSCs in cocultures, with and without chloroquine-treatment. Each dot indicates the cell proliferation of AML or MSCs after coculture, where results are shown as percent proliferation of chloroquine-treated cocultures compared to untreated cocultures, with the median level. Chloroquine inhibited the proliferation of both AML cells and MSCs in cocultures.

We further investigated the antiproliferative effect of chloroquine on AML cells when the leukemic cells were cocultured in the presence of normal MSCs derived from a healthy donor. The two cell populations were separated by a semipermeable membrane where direct contact between AML cells and MSCs was not possible; the 3H-thymidine incorporation assay was then used to measure proliferation of cells in cocultures after incubation with or without 5 µM chloroquine for three days. Of the 18 patients tested, 14 showed detectable AML cell proliferation in the medium controls (cpm > 1000). As expected, MSCs increased AML cell proliferation for the majority of patients (data not shown) [31]. Still, AML cell proliferation was decreased after treatment with 5 µM chloroquine in cocultures for most patients (*p*-value = 0.017, Wilcoxon signed rank test) (Figure 3). Chloroquine also inhibited MSC proliferation (derived from one donor), and this inhibitory effect on MSCs was stronger than the effect on the AML cells (Figure 3).

### 3.6. An Antiproliferative Effect of Chloroquine Is Detected for Most Patients and Even for Patients Insensitive to Cytarabine

**Figure 4:**
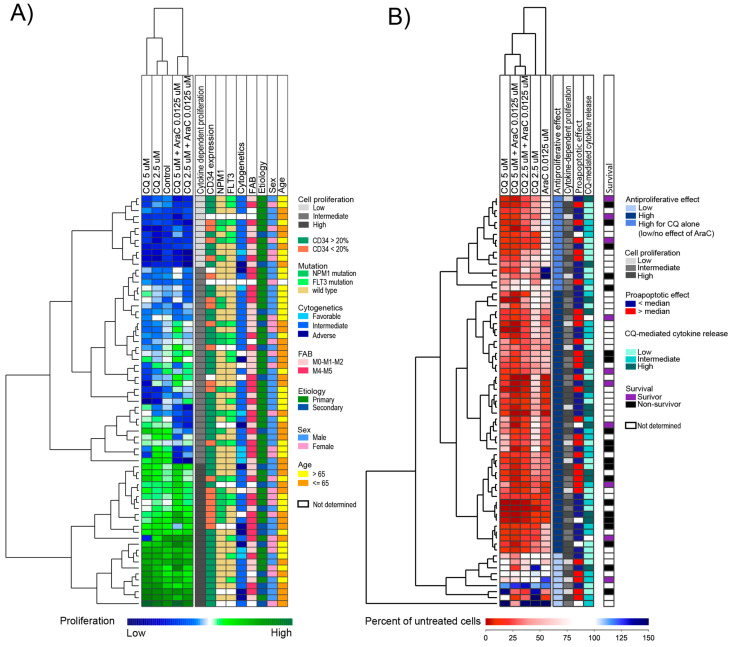
An unsupervised hierarchical cluster analysis based on the effect of chloroquine and cytarabine/AraC on AML cell proliferation. AML cells from 81 consecutive patients were cultured for seven days with chloroquine (CQ 2.5 and 5 µM), AraC (0.0125 µM), chloroquine in combination with AraC or medium alone (control). Proliferation was measured using a 3H-thymidine incorporation assay. Detectable proliferation was defined as >1000 cpm, and results are presented for the 69 patients with detectable proliferation in untreated cultures. (**A**) The figure illustrates the cytokine-dependent AML cell proliferation for the untreated controls and drug-treated cultures (chloroquine or a combination of drugs) after results were normalized to the corresponding median for each group. The cluster could be divided into three main subsets based on the degree of proliferation as illustrated in the first column to the right: (i) low proliferation (upper cluster, light gray), (ii) intermediate proliferation (middle, gray), and (iii) high proliferation (lower cluster, dark gray). The figure also shows the distribution of biological and clinical characteristics for each individual patient (columns on the right part of the figure). (**B**) The figure shows the relative AML cell proliferation (i.e., percent proliferation compared to untreated controls) for the 69 AML patients after treatment with chloroquine and AraC. As shown, the majority of patients had a strong inhibitory effect of chloroquine, AraC or both drugs (two top subclusters, shown as blue and dark blue in the column to the right). A small subcluster of nine patients (bottom subcluster, shown as light blue in the column to the right) had mainly little or no effect of these treatments at the tested concentrations. Shown in different columns to the right of the figure are different patient subsets based on clustering of cytokine-dependent proliferation (patient classification as indicated in Figure 4A), proapoptotic effects (classified based on Figure 5, see Section 3.8), chloroquine-mediated cytokine release (based on Figure 6, see Section 3.9), and survival after completed intensive treatment. Survival is presented only for patients who completed the planned intensive and consolidation treatment, and all patients classified as survivors were observed for at least three years after treatment.

We performed an unsupervised hierarchical cluster analysis where we compared the AML cell proliferation (i.e., normalized to the median cpm value for each group) after cells had been cultured in medium alone, in the presence of chloroquine or after combined treatment with chloroquine and cytarabine for 7 days. In this analysis, we only included the 69 patients demonstrating detectable proliferation (>1000 cpm) in untreated control cultures. We could then detect three patient subsets: a small subset with generally low proliferation in untreated cultures as well as in drug-treated cultures (upper subcluster, indicated by the light gray column in Figure 4A), a larger subset with generally strong proliferation in both treated and untreated cultures (bottom green subcluster, shown as a dark gray column in Figure 4A), and an intermediate subset with diverse proliferation in both untreated and drug-treated cultures (middle subcluster, indicated as a gray column in Figure 4A). 

Next, we performed an unsupervised hierarchical clustering analysis based on the relative proliferation of the drug-treated cell cultures, i.e., the proliferation in drug-treated cultures (chloroquine or cytarabine) relative to the proliferation in the control cultures prepared in medium alone after seven days of culture (Figure 4B). Patients showed a varied response to drug treatment. Effects on cell proliferation after treatment with either chloroquine 5 µM or cytarabine (0.0125 µM) alone ranged from 0–99% reduced proliferation, with a median reduction of 83% and 58% compared to control cultures, respectively. Sixty-two of the 69 patients treated with chloroquine 5 µM, and 58 of the 69 patients treated with chloroquine 2.5 µM demonstrated an antiproliferative effect (>20% reduction of proliferation compared to untreated controls), whereas the last nine patients had no or minor effects of both drugs (Figure 4B, lower patient sub-cluster). However, we also identified a subset of patients that were sensitive towards chloroquine (strong antiproliferative effect), although no or low inhibitory effects on cell proliferation were seen after treatment with cytarabine alone (Figure 4B, the upper 16 patients).

The antiproliferative profile of chloroquine (i.e., the classification into three clusters in Figure 4B) showed no significant associations with age, cause of AML (de novo versus secondary), morphological signs of differentiation (FAB classification), expression of the stem cell marker CD34, karyotype, or *FLT3*-ITD or *NPM1* mutations (data not shown). Finally, the relative effect of chloroquine/cytarabine also showed no association with the capacity of cytokine-dependent proliferation (Figure 4A).

### 3.7. The Antiproliferative Effect of Chloroquine on AML Cells Is Associated with a Distinct Gene Expression Profile

Based on the antiproliferative effects of chloroquine 2.5 µM we created a heat map, which sorted the patients according to the antiproliferative effects on AML cell proliferation compared to control cultures (Figure S2, upper part). The heat map indicates a strong antiproliferative effect to the left (high sensitivity to chloroquine) and patients with the lowest antiproliferative effect of chloroquine to the right (low sensitivity to chloroquine). Based on this sorting we divided the patient cohort into four quartiles, and compared gene expression profiling (GEP) data for randomly selected patients from the lowest and highest quartiles; i.e., patients with strong antiproliferative effects and patients with low or no antiproliferative effects of chloroquine. GEP data was available for six patients with high sensitivity to chloroquine, and 11 patients with low sensitivity to chloroquine treatment (marked in boxes). Based on ANOVA (F-score > 1.0 and FC value > 1.0), we identified 99 genes upregulated among patients with high sensitivity (Table S5). Among the 99 identified genes, 22 have previously been linked to AML leukemogenesis, and the full list with references to AML involvement is presented in Table S5. Furthermore, we did an unsupervised hierarchical cluster analysis based on these 99 genes, but this analysis could not be used to separate patients with high and low sensitivity to chloroquine. Finally, we used the PANTHER system to further classify the upregulated genes, and we then selected the category Molecular Function which included Catalytic activity and Binding as the largest subterms within this category (Figure S2, lower part). We further identified single genes belonging to these subterms (Figure S2 lower part, see also Table S5).

### 3.8. Treatment with Chloroquine Significantly Decreased Primary AML Cell Viability and Increased Apoptosis and Necrosis

**Figure 5:**
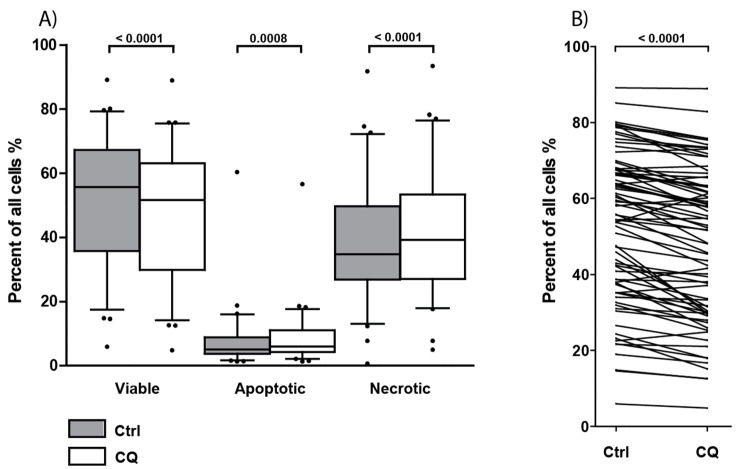
Effect of chloroquine (CQ) 5 µM on primary AML cell viability, apoptosis, and necrosis after 48 h of culture. Primary cells from 78 consecutive AML patients were cultured with or without 5 µM CQ for 48 h before viability, early apoptosis, and late apoptosis/necrosis were determined by flow cytometry using the AnnexinV/Propidium iodide (PI) assay. (**A**) The figure shows the overall results for the 72 patients with more than 5% viable cells in drug-free controls. The percentage of viable (AnnexinV^−^ PI^−^), early apoptotic (AnnexinV^+^ PI^−^) and end stage apoptotic and necrotic cells (AnnexinV^+^ PI^+^) were determined in patient samples after treatment with 5 µM CQ (white boxes) and after culture in medium alone (gray boxes) for 48 h. Data are presented as median levels, 25/75 percentiles, and 5/95 percentile whiskers, • = outliers. The overall effect when analyzing samples pairwise was examined; and treatment with 5 µM CQ significantly decreased viability (*p*-value < 0.0001) and increased apoptosis and late apoptosis/necrosis (*p*-values = 0.0008 and <0.0001, respectively; Wilcoxon signed rank test). (**B**) The percentage of viable primary AML cells cultured in medium alone compared to treatment with 5 µM CQ for 48 h. This figure presents the viability results for each individual patient. A wide range of cell viability is seen among patients, with a significant decrease in viability after treatment with 5 µM CQ (*p*-value = 0.0001).

Primary cells derived from 78 of the 81 consecutive AML patients were cultured with or without chloroquine 5 µM for 48 h before the percentages of viable, early apoptotic, and late apoptotic/necrotic cells were determined by flow cytometry. Six patients showed less than 5% viable cells in untreated controls and were excluded from the statistical analyses. There was a wide variation between patients with regard to the percentage of viable (AnnexinV^−^PI^−^) cells in drug-free control cultures, with only a slight decrease in overall viability after chloroquine treatment (median 52.0%, range 4.8–88.9%) compared with the medium controls (median 55.7%, range 5.9–89.1%) (Figure 5A). The percentage of early apoptotic cells (AnnexinV^+^PI^−^) was generally low for both treated and untreated cultures, with a slight increase in apoptosis after chloroquine treatment (Figure 5A). When comparing samples pairwise, there was a small but statistically significant decrease in the percentage of viable cells after chloroquine treatment (*p*-value < 0.0001, Wilcoxon signed rank test) and an increase in the percentage of early apoptotic cells (*p*-value = 0.0008, Wilcoxon signed rank test). The effect of 5 µm chloroquine on AML cell viability was relatively weak, certain exceptional patients showed a small increase in the percentage of viable AML cells, and patients with a high viability in untreated control cultures generally showed the highest viability also in the presence of the drug (Figure 5B, *p*-value = 0.0001, Wilcoxon signed rank test). Thus, chloroquine has a weak but statistically significant effect on AML cell viability, and this reduction seems to be caused by induction of apoptosis.

Neither the AML cell viability in control cultures nor the effect of chloroquine on AML cell viability showed significant associations with age, cause of AML (de novo versus secondary), morphological signs of differentiation (FAB classification), expression of CD34, karyotype, *FLT3*-ITD, or *NPM1* mutations (data not shown). This is similar to the results when investigating the effect of chloroquine and the antiproliferative profiles (Figure 4) described previously.

### 3.9. Chloroquine Alters the Constitutive AML Cell Release of Only a Few Soluble Mediators by Primary Human AML Cells

The effect of chloroquine on the constitutive soluble mediator release by primary AML cells after 48 h of culture was investigated for the same 72 patients tested in the viability assay. No significant changes were found between mediator levels when comparing overall results, but when comparing pairwise samples (untreated versus chloroquine-treated), a significant effect of chloroquine on the release of mediators was observed for four mediators, MMP9, MMP2, cystatin-C, and CCL2 (Figure S3; *p*-value < 0.05, Wilcoxon signed rank test).

**Figure 6:**
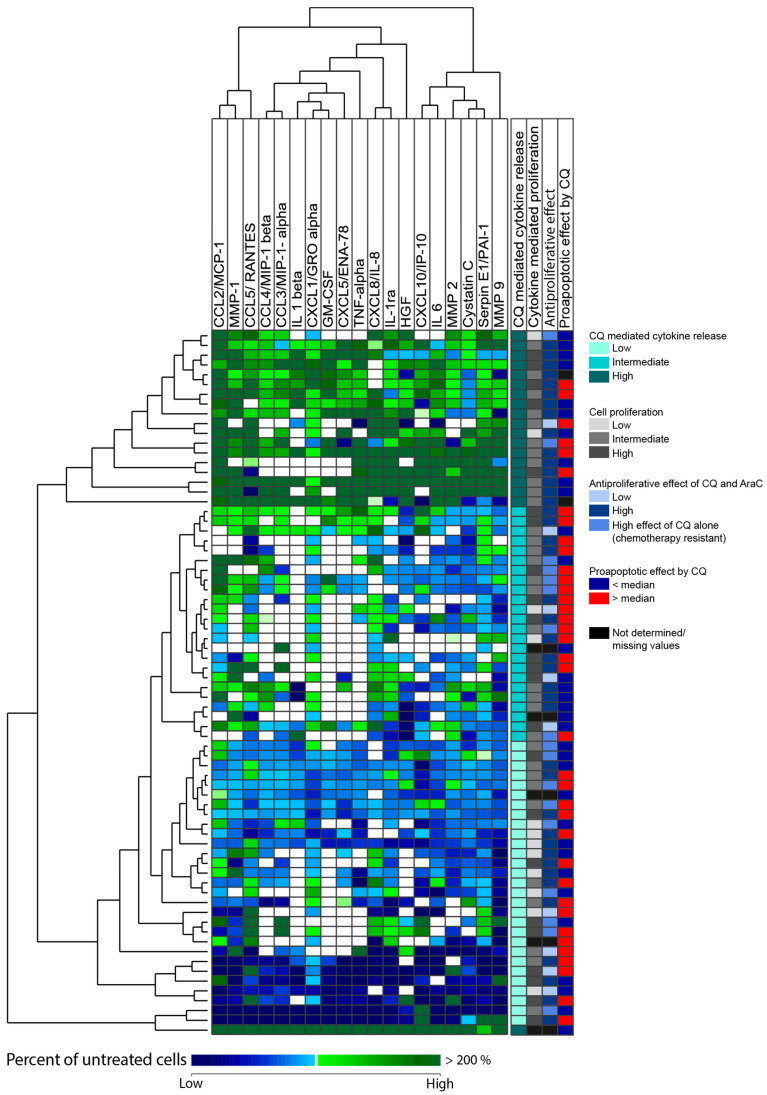
(Page 15). Unsupervised hierarchical cluster analysis based on alterations in the release of soluble mediators by primary AML cells derived from 72 unselected patients after treatment with 5 µM chloroquine (CQ) for 48 h. Primary AML cells were cultured with 5 µM chloroquine or medium alone for 48 h, before the levels of soluble mediators in harvested supernatants were determined using Luminex analysis. A hierarchical cluster analysis was performed based upon the alteration of soluble mediators compared to untreated controls (control set to 100%). The cluster analysis identified three patient subsets: (i) a subset with increased release of soluble mediators after treatment with chloroquine (upper subset, shown as dark green in first column to the right), (ii) one intermediate subset, with divergent release of soluble mediators (middle subset, shown as turquoise in column to the right), and (iii) a subset of patients with decreased release of soluble mediators after treatment with chloroquine (light turquoise). There is one outlier at the bottom of the cluster with a very high release of all soluble mediators after treatment with chloroquine (marked in dark green). The columns to the far right indicating proliferative responses and chloroquine effects are based on Figure 4.

Furthermore, we performed a hierarchical cluster analysis that identified a subset of 18 patients where chloroquine generally increased the levels of various soluble mediators, whereas chloroquine had divergent effects for the other patients with unaltered or decreased levels after chloroquine treatment for most mediators and patients (Figure 6). The three main subsets identified in the cluster analysis showed no significant differences with regard to age, cause of AML (de novo versus secondary), morphological signs of differentiation (FAB classification), expression of the CD34 stem cell marker, karyotype, *FLT3*-ITD, or *NPM1* mutations (data not shown). However, we identified a patient cluster including 18 patients that showed a very high release of mediators, and this subset included a significantly higher fraction of patients showing a strong antiproliferative effect after treatment with both chloroquine 2.5 μM and cytarabine (i.e., 15 out of the 18 patients being susceptible to both drugs, see Figure 4B) compared with the other patients (25 out of 48 patients showing dual effects, Fisher’s exact test, *p* = 0.0252). 

### 3.10. High Chloroquine-Mediated Soluble Mediator Release Shows a Gene Expression Profile Associated with Genes Involved in Metabolic Processes

Gene expression profiling (GEP) data was available for a total of 33 patients that were examined for their release of soluble mediators after chloroquine treatment. Nine of these 33 patients belonged to the previously identified group of patients with high chloroquine-mediated mediator release, while the remaining 24 belonged to the group of patients with intermediate or low cytokine release (see Figure 6). Using ANOVA (F-score > 1.0 and FC value > 1.0) we identified 74 genes upregulated among patients with high chloroquine-mediated mediator release (Table S6). 

We investigated if these 74 identified genes were able to separate the two patient groups by performing a hierarchical cluster analysis. Only two of the nine patients with high chloroquine-mediated mediator release clustered outside the identified main subcluster including most patients with high chloroquine-induced mediator release (*p*-value = 0.001, Fisher´s exact test) (Figure S4). Among the 74 genes that were upregulated for patients with high chloroquine-mediated release of various mediators were several genes encoding proteins involved in AML pathogenesis. All genes are presented in Table S6. To investigate a broader biological context of the upregulated genes, we used the PANTHER classification system to further classify these genes. The four most frequent subterms were Metabolic process, Cellular process, Localization, and Response to stimulus when using the main category Biological process. We further identified single genes belonging to these subterms, which included genes known to be involved in AML leukemogenesis, noteworthy, *SNX2*, *FLT3*, *PFKP*, and *CCL23* (Figure S4, Table S6).

## 4. Discussion

The long-term AML-free survival is only 45–50% even for patients who receive the most intensive antileukemic treatment [4]. Thus, there is a need for new therapeutic strategies to increase the efficiency of conventional intensive treatment and to prolong survival for elderly/unfit patients receiving AML-stabilizing treatment. Inhibition of autophagy, e.g., by chloroquine or chloroquine analogs, is regarded as a possible approach for treatment of cancer, including AML [21,22,24,41,42], but our present results suggest that the susceptibility to chloroquine varies between patients.

Chloroquine inhibits lysosomal acidification and thereby arrests the last step of autophagy i.e., fusion of autophagosomes with lysosomes [21,42], but it may also have additional effects including (i) increased lysosomal permeability with intracellular release of proteolytic enzymes [24], (ii) inhibition of cellular drug-extrusion, certain lysosomal enzymes and intracellular signaling, and (iii) intercalation into DNA [43,44,45,46]. Thus, anticancer effects of chloroquine can be mediated by various mechanisms, including inhibition of autophagy, but at least in our present study, we did not find any evidence for altered levels of proteins belonging to the autophagy-associated ULK1, PI3K, Atg9, and Atg12 complexes.

Previous AML studies have mainly investigated effects of chloroquine in cell lines [13,47,48,49], and then, many of chloroquine’s antileukemic effects depend on inhibition of autophagy, e.g., inhibition of dasatinib-induced differentiation [47], apoptosis induction in cytarabine-sensitive and resistant cell lines [48], caspase-dependent apoptosis in erythroleukemia with downregulation of c-Myc, upregulation of proapoptotic gene expression and modulation of the cellular miR profile in favor of apoptosis [49]. Furthermore, chloroquine-induced inhibition of autophagy enhances the antileukemic effects of cytarabine [20,50] and mTORC1/mTORC2 inhibitors [25]. Additionally, autophagy-independent antileukemic effects of chloroquine in AML possibly include (i) modulation of exocytosis [13], (ii) induction of hypoxia-inducible factor 1α [51], (iii) modulation of cellular iron metabolism [52,53] and (iv) unwinding of the DNA double helix [54]. However, although the effects are complex and differ between various cell types, these previous studies together with our present results show the importance of autophagy inhibition by chloroquine. Our present study is the first to show susceptibility to chloroquine for a large group of unselected AML patients. We describe an antiproliferative effect of chloroquine; this effect was dose-dependent and when testing relatively low concentrations it varied between patients. However, an antiproliferative effect was also detected for all four UCB donors and for MSCs; and such effects on normal stem cells may be involved in the development of hematological toxicity that has been reported in exceptional patients, including leukopenia and agranulocytosis [55].

We used drug concentrations corresponding to the levels reached in vivo during long-term chloroquine treatment (plasma levels 2.5–12.5 μM) [56,57]. In addition, we tested higher concentrations that are closer to what is seen during treatment of malaria (25–440 µM over three days) [58]. The cytarabine levels tested in our study correspond to serum concentrations reached during low-dose subcutaneous cytarabine therapy [59] and are thus also within clinically relevant concentrations [60].

We included only patients with relatively high levels of circulating AML cells, and therefore enriched leukemia cell populations could be prepared by standardized density gradient separation alone [61,62], thereby reducing the risk of separation-induced cellular alterations [63]. For this reason, our observations should be interpreted with caution as they may be representative only for patients with relatively high peripheral blood blast counts, though our results are probably representative for bone marrow AML cells, as blood and marrow AML cells do not differ with regard to autophagy [23].

Our proliferation assay was based on [3H]-thymidine incorporation from day six to seven of in vitro culture, i.e., the incorporation reflects characteristics of the minor cell subset that is able to survive and proliferate after seven days of culture [35]. Our results showed that chloroquine had a significant and dose-dependent effect on AML cell proliferation for the large majority of patients, though the inhibitory effect varied between patients and was even absent for a small minority. Furthermore, chloroquine showed an additional antiproliferative effect in the presence of cytarabine, and this effect was seen even for cytarabine-resistant cells. These last observations are consistent with previous reports describing that AML cells utilize autophagy to counteract chemotherapeutic-induced stress, and blocking autophagy can then enhance sensitivity to cytotoxic drugs [19]. However, the antiproliferative and proapoptotic effects of chloroquine showed no significant correlation, suggesting that they are independent pharmacological effects. Finally, previous studies have demonstrated that biological characteristics of the total AML cell populations can reflect the relapse risk [64,65,66,67,68,69,70,71], i.e., the chemosensitivity of the leukemic stem cells (LSCs) responsible for relapse [64], and our present results may thus be representative also for LSCs, though further studies are needed to investigate specific effects on LSCs.

Basal levels of autophagy vary among AML cell lines and there is also a variation between patients; a higher autophagic flux was described for AML patients with complex karyotype whereas no differences were detected in AML with recurrent genetic abnormalities with prognostic value [23]. We also did not find any associations between the effects of chloroquine and established prognostic parameters. Previous studies have shown that the prognostic impact of a biomarker can depend on the clinical/biological context [72], and this may explain the lack of associations between sensitivity to chloroquine and established prognostic biomarkers in AML. Thus, the use of chloroquine in AML therapy possibly needs to be individualized based on the identification of new and validated biomarkers that can identify potential responders to this specific treatment. Our present proteomic studies suggest that selected proteins expressed by enriched AML cells may be useful as potential biomarkers for susceptibility to chloroquine, and flow cytometric protein expression analysis of the AML cell population would then be a methodological approach that is suitable for routine practice.

Bafilomycin 1A is a V-ATPase inhibitor that blocks the fusion between autophagosomes and lysosomes [73,74]; chloroquine and bafilomycin 1A thus have common cellular pharmacological effects despite having distinct molecular mechanisms. There was a significant association between the antiproliferative effects of chloroquine and bafilomycin; an observation suggesting that their common pharmacological effect on lysosomes/autophagy is important for their antiproliferative activity in primary AML cells.

Analysis of the overall results showed that chloroquine decreased the constitutive extracellular release of MMP-2, MMP-9, and cystatin-C whereas CCL2 levels increased. Previous studies have also demonstrated that chloroquine affects the matrix metalloproteinase network [75] and can induce chemokine expression through NFκB activation [76]. However, the chloroquine effect on constitutive mediator release differed between individual patients. First, a general increase in soluble mediator release was observed for a subset of patients, whereas the other patients showed unaltered or decreased levels for most mediators. Second, we examined the global gene expression profiles for 33 patients; nine of which showed a generally higher mediator release after chloroquine exposure, and these patients showed leukemic cell upregulation of several genes (*SNX2*, *FLT3*, *PFKP*, and *CCL23*) involved in AML leukemogenesis (Figure S4). Third, a wide variation between patients in their constitutive release profiles was maintained even after exposure to chloroquine (Figure S3). Thus, chloroquine can alter communication between AML cells and neighboring AML-supporting non-leukemic cells and thereby have indirect effects on the stromal cells in addition to its direct effects [17,77].

Chloroquine can have adverse effects on many cells and organs, including normal hematopoietic cells [24]. Our present results show that chloroquine has antiproliferative effects on both mononuclear UCBs and MSCs. Future clinical studies must address the question of toxicity, especially hematological toxicity that often is dose-limiting in AML therapy [1,3,4] and is particularly important for elderly patients with age-dependent stem cell defects that become visible during stress, e.g., hematological regeneration after cytotoxic anticancer treatment [78]. Possible strategies to increase anticancer efficacy and/or decrease toxicity by chloroquine are the use of chloroquine analogs/hybrids or nanoparticles for targeted delivery [79,80,81].

Several new therapeutic strategies targeting specific molecular mechanisms are emerging in AML, including kinase, IDH, and BCL2 inhibitors [1] together with inhibition of autophagy. These approaches are also considered for combination therapy together with intensive and potentially curative treatment, conventional disease-stabilizing therapy [1] or new targeted therapies. Venetoclax combined with a demethylating agent or low-dose cytarabine should probably be preferred for the treatment of AML in elderly and unfit patients [82,83,84,85,86,87,88,89]. It will therefore be important to investigate both the efficacy and the toxicity if chloroquine or other autophagy inhibitors are combined with venetoclax alone or venetoclax plus a cytarabine/demethylating agent in future clinical studies.

Personalized or precision AML treatment is now regarded as a possible therapeutic strategy in AML [90]. Our present study included a limited number of molecular genetic markers. Future studies should try to clarify the possible role of autophagy inhibition (e.g., chloroquine therapy) in future personalized AML therapy, i.e., whether inhibition of autophagy is more effective for certain subsets of patients, and try to identify genetic and/or proteomic biomarkers for susceptibility to autophagy inhibition.

## 5. Conclusions

Our study shows that direct antileukemic effects of chloroquine on AML cells were observed for most patients, but only a subset of patients was highly sensitive to the drug when tested at low concentrations. Furthermore, chloroquine seems to have additional indirect effects on AML cells mediated by bone marrow stromal cells and also altered the release of soluble mediators. Finally, there is a risk of direct toxicity of chloroquine against normal cells, including bone marrow toxicity. Our present results suggest that chloroquine or chloroquine analogs should be further explored in AML, but future clinical studies should focus on patient heterogeneity and identification of (protein) biomarkers that are associated with chloroquine sensitivity. Personalized or precision therapy is now considered in AML [90]. If chloroquine could be used in selected patients, as personalized medicine, one would expect in vivo concentrations to have increased effectiveness with a lower risk of severe dose-dependent toxicity.

## Figures and Tables

**Table 1:** A classification of proteins that show significantly different levels when comparing AML cells with strong and weak antiproliferative effects of chloroquine. The gene names are shown to the right. The proteins are described in detail in Table S3 and the results of the proteomic analyses are summarized in Table S4.

Classification	Proteins (Referred to by Their Corresponding Gene Names)
Autophagy regulation	*SIGIRR*, *PGPEP1*, *STK38L*, *DAP3*, *YBX1*, *CSDE1*, *PRMT1*, *HGSNAT*, *FAF1*, *FAM105A*
Mitophagy regulation	*ATPIF1*, *PGPEP1*, *STK38L*
Cytoskeletal protein	*DNAJC1*, *SIGIRR*, *TUBA1A*, *NUDCD3*, *TUBB6*, *TPPP3*
Intracellular trafficking	*SYTL1*, *WDR81*
Endoplasmic reticulum	*WDR81*, *LEPRE1*, *DPM3*, *CALU*, *SERPINH1*, *LY75*
Lysosomal protein	*WDR81*, *LY75*, *HGSNAT*
Mitochondria, metabolism	*SARDH*, *SLC2A5*, *ATPIF1*, *H6PD*, *DAP3*, *MMS19*, *HK2*
Extracellular release	*PPBP*, *YBX1*
Cell surface/adhesion	*HLA-E*, *EPB41L2*, *ITGB3*, *ITGA2B*, *DPYSL3*
Intracellular signaling	*DNAJC1*, *SIGIRR*, *STK38L*, *FAF1*, *TSTD1*
Transcription	*SUGP2*, *SAP30L*, *PGPEP1*, *GTF2E2*, *NPM3*, *CRIP2*

## Data Availability

Data is contained within the article or supplementary material. Additional data may be available upon request to interested researchers.

## Supplementary Materials

The following are available online at https://www.mdpi.com/article/10.3390/jpm11080779/s1, Table S1. Clinical and biological characteristics of the AML patients included in the present study. Table S2. Mutational analyses and the antiproliferative effects of chloroquine. Table S3. A description of proteins with significantly different levels when comparing AML cells showing strong and weak antiproliferative effects by chloroquine treatment. Table S4. A description of proteins that show significantly different levels when comparing AML cells showing strong and weak antiproliferative effects of chloroquine, a summary of the bioinformatical analyses. Table S5. The complete list of 99 significantly increased genes (F-score > 1.0 and FC value > 1.0) for patient samples with high sensitivity to chloroquine. Table S6. The complete list of 74 significantly increased genes (F-score > 1.0 and FC value > 1.0) in patient samples with upregulated release of soluble mediators after chloroquine treatment. Figure S1. The inhibitory effect of chloroquine (CQ) on autophagy in AML cell lines. Figure S2. Profiling of gene expression data based on antiproliferative effects of chloroquine on primary AML cells. Figure S3. Effect of chloroquine treatment on the constitutive release of soluble mediators in AML cell cultures and in AML-MSC cocultures. Figure S4. Gene expression profiles associated with levels of soluble mediators released by AML cells after chloroquine treatment.
