# Supplementary material for: Effects of the Autophagy-Inhibiting Agent Chloroquine on Acute Myeloid Leukemia Cells; Characterization of Patient Heterogeneity
Source: J Pers Med. 2021 Aug 10;11(8):779. doi: 10.3390/jpm11080779 (PMC8399694; doi:10.3390/jpm11080779)
Supplement: Supplementary file 1 [file jpm-11-00779-s001.zip › jpm-1319362-supplementary.pdf]

Effects of the autophagy-inhibiting agent chloroquine on acute myeloid leukemia cells; characterization of patient heterogeneity

Ida Sofie Grønningsæter [1,2], Håkon Reikvam [1,3], Elise Aasebo [1,4], Sushma Bartaula-Brevik [1], Maria Hernandez-Valladares [4,5], Frode Selheim [4,5], Frode S. Berven [4,5], Tor Henrik Tvedt [3,6], Øystein Bruserud [1,3]* and Kimberley Joanne Hatfield [1,7]*

[1] Department of Clinical Science, University of Bergen, N-5021 Bergen, Norway;

[2] Department of Medicine, Akershus University Hospital, Oslo, Norway;

[3] Department of Medicine, Haukeland University Hospital, N-5021 Bergen, Norway;

[4] The Proteomics Facility of the University of Bergen (PROBE), University of Bergen, Bergen, Norway;

[5] The Department of Biomedicine, University of Bergen, Bergen, Norway;

[6] Department of Hematology, Oslo University Hospital – The National Hospital, N-0372 Oslo, Norway;

[7] Department of Immunology and Transfusion Medicine, Haukeland University Hospital, Bergen, Norway;

* Correspondence: Oystein.Bruserud@helse-bergen.no, Tel: +47 55972997, Fax: +47 55972950; Kimberley.Joanne.Hatfield@helse-bergen.no, Tel.: +47 55973037

Table S1. Clinical and biological characteristics of the AML patients included in the present study. **Table S2.** Mutational analyses and the antiproliferative effects of chloroquine. **Table S3.** A description of proteins with significantly different levels when comparing AML cells showing strong and weak antiproliferative effects by chloroquine treatment. **Table S4.** A description of proteins that show significantly different levels when comparing AML cells showing strong and weak antiproliferative effects of chloroquine, a summary of the bioinformatical analyses. **Table S5.** The complete list of 99 significantly increased genes (F-score >1.0 and FC value >1.0) for patient samples with high sensitivity to chloroquine. **Table S6.** The complete list of 74 significantly increased genes (F-score >1.0 and FC value >1.0) in patient samples with upregulated release of soluble mediators after chloroquine treatment. **Figure S1.** The inhibitory effect of chloroquine (CQ) on autophagy in AML cell lines. **Figure S2**. Profiling of gene expression data based on antiproliferative effects of chloroquine on primary AML cells. **Figure S3.** Effect of chloroquine treatment on the constitutive release of soluble mediators in AML cell cultures and in AML-MSC cocultures. **Figure S4.** Gene expression profiles associated with levels of soluble mediators released by AML cells after chloroquine treatment.

Table S1. **Clinical and biological characteristics of the AML patients included in the present study.**

CLINICAL CHARACTERISTICS					
Gender			*Age*		
Female	30		Median (years)		
Male	51		Range (years)	67.5<br>18-87	
Predisposition/previous hematological disease			*Long-term AML-free survival after intensive therapy* (i.e. all patients observed for at least 3 years)		
AML relapse	5		Yes	15	
MDS	11		No	24	
CMML	2		No potential curative therapy	42	
Polycythemia Vera	1				
Li-Fraumeni syndrome	1				
**AML CELL DIFFERENTIATION**					
FAB classification			*CD34 expression*		
AML-M0/M1/M2	38		Negative (<20%)	22	
AML-M4/M5	36		Positive (>20%)	52	
nd	7		nd	7	
**GENETIC ABNORMALITIES**					
Cytogenetics		*FLT3 abnormalities*		*NPM1 abnormality*	
Normal	37	wt	45	wt	45
Favorable	10	ITD	22	INS	28
Intermediate	11	TKD	2	nd	8
Adverse	14	ITD/TKD	3		
nd	9	nd	9		

Abbreviations: CMML, chronic myelomonocytic leukemia; FAB, French-American-British; INS, insertion; ITD, internal tandem duplication; MDS, myelodysplastic syndrome; nd, not determined; TKD, tyrosine kinase domain; wt, wild type.

Table S2. Mutational analyses and the antiproliferative effects of chloroquine. Additional mutational analyses of 54 genes were available for an unselected subset of consecutive AML patients unfit for intensive therapy (including hypomethylating agents). The clinical and biological characteristics of the 15 patients are shown, including the genetic analyses and the effect of chloroquine (second column) on cytokine-dependent *in vitro* proliferation. The patients are ranked based on the antiproliferative effect of chloroquine 2.5 µM, i.e. the percentage of AML cell proliferation in cultures treated with chloroquine compared to corresponding control cultures prepared in medium alone.

**Id**	**Effect of chloroquine**	**Gender**	**Age**	**Predispo-sition**	**FAB**	**Membrane molecule expression**					**Karyotype**	***FLT3***	***NPM1***	**Additional mutations**	**WBC counts (x 10^9/L)**
	(%)					CD13	CD14	CD15	CD33	CD34					
1	135	F	77	MDS	M2	+	-	-	+	+	normal	wt	wt	*NRAS, TET2, ASXL1, RUNX1, SRSF1, STAG2, BCDR*	142
2	111	F	70	MDS	M2	nt	nt	+	+	+	del12	wt	wt	*NRAS, KRAS, PTPN11, ASXL1, STAG2*	81.0
3	88	M	74	*de novo*	M0	+	-	-	+	+	multiple	wt	wt	*TP53*	18.7
4	70	M	68	Myelo-fibrosis	M1	+	-	-	+	+	normal	wt	wt	*KRAS*	34.3
5	68	M	48	relapse	M4	+	nt	-	+	+	normal	ITD, TKD	INS	*DNMT3A, IDH1*	30.4
6	60	F	78	*de novo*	M0	+	-	-	-	+	nt	wt	wt	*PTPN11, ASXL1, RUNX1, SRSF2*	21.0
7	55	F	60	relapse	M4	+	-	+	+	-	normal	ITD	INS	*DNMT3A, TET2*	16.7
8	47	M	73	*de novo*	M1						nt	wt	INS	*TKD, IDH2, SRSF1*	12.1
9	42	M	71	Chemo-therapy	M4	+	-	-	+	+	normal	wt	INS	*KRAS, DNMT3A, TET2*	104
10	40	M	74	*de novo*	M0	+	-	-	-	+	multiple	wt	wt	*IDH2*	13.3
11	34	M	67	1st relapse	M1	+	-	-	-	+	normal	TKD	wt	*None*	15.6
12	31	F	77	*de novo*	M1	-	-	-	-	-	normal	ITD	INS	*DNMT3A*	68.5
13	30	F	57	Li Fraumeni	M0	+	-	-	+	-	multiple	wt	wt	*TP53*	36.9
14	17	F	77	*de novo*	M2	+	-	-	+	-	normal	ITD	INS	*DNMT3A*	77.8
15	3	M	60	2nd relapse	M4	+	-	+	+	+	normal	ITD	wt	*WT1*	66

Abbreviations: F, female; FAB, French-American-British; INS, insertion; ITD, internal tandem duplications; M, male; MDS, myelodysplastic syndrome; nt, not tested; TKD, tyrosine kinase domain; WBC, white blood cell; wt, wild type.

Table S3. A description of proteins with significantly different levels when comparing AML cells showing strong and weak antiproliferative effects by chloroquine treatment.

Gene names	Protein names	
ITGB3	*Integrin beta-3.* The protein product is the integrin beta chain beta 3. Integrins are integral cell-surface proteins composed of an alpha chain and a beta chain. Integrins participate in cell adhesion as well as cell-surface mediated signalling.	Adhesion<br>Cell signaling
ITGA2B	*Integrin alpha-IIb.* The protein product is the integrin alpha chain II b. Integrins are integral cell-surface proteins composed of an alpha chain and a beta chain. Integrins participate in cell adhesion as well as cell-surface mediated signalling.	Adhesion<br>Cell signaling
SARDH	*Sarcosine dehydrogenase, mitochondrial.* The protein is important in sarcosine metabolism.	Mitochondria.<br>Metabolism.
PPBP	*Pro-platelet basic protein.* The protein is a growth factor that belongs to the CXC chemokine family. It is a potent chemoattractant and activator of neutrophils. It has been shown to stimulate various cellular processes including DNA synthesis, mitosis, glycolysis, intracellular cAMP accumulation, prostaglandin E2 secretion, and synthesis of hyaluronic acid and sulfated glycosaminoglycan.	CXC chemokine
HLA-E	*HLA class I histocompatibility antigen, alpha chain E.* HLA-E belongs to the HLA class I heavy chain paralogues. This class I molecule is a heterodimer consisting of a heavy chain and a light chain (beta-2 microglobulin). The heavy chain is anchored in the membrane.	Plasma membrane
SLC2A5	*Solute carrier family 2, facilitated glucose transporter member 5.* The encoded protein is a fructose transporter.	Metabolism
SYTL1	*Synaptotagmin-like protein 1.* The gene encodes a trafficking protein that controls Rac1-GTP recycling (PMID 30748033), it is also aregulator of intracellular vesicular transport (PMID 27701149). The protein determines intracellular trafficking of CXCR4 and thereby promotes migration of malignant myeloid cells via activation of the CXCL12/CXCR4 axis (PMID 27018596.	Intracellular transport<br>CXCL12/CXCR4
TTN	*Titin.* This protein is a part of the cytoskeleton in muscles but has also been identified as a structural protein for chromosomes.	Chromosome
SUGP2	*SURP and G-patch domain-containing protein 2.* This gene encodes a member of the arginine/serine-rich family of splicing factors. The encoded protein functions in mRNA processing.	RNA processing
WDR81	*WD repeat-containing protein 81.* This gene encodes a multi-domain transmembrane protein which is predominantly expressed in the brain and is thought to play a role in endolysosomal trafficking.	Endolysosomal trafficking
SAP30L	*Histone deacetylase complex subunit SAP30L.* The protein is involved in transcriptional regulation (PMID 22821512), its structure and function is regulated by the redox balance (PMID 26609676), its is important for targeting of the Sin3A complex to the nucleolus (PMID 16820529) and is a TGFβ target gene (PMID 14680513).	Nucleolus<br>Transcription<br>TGFβ
DNAJC1	*DnaJ homolog subfamily C member 1.* The encoded membrane protein is a DNAJ-like heat shock protein that binds the molecular chaperone HSPA5 (heat shock protein family A (Hsp70) member 5). In addition, the encoded protein contains two SANT domains that have been shown to bind serpin alpha1-antichymotrypsin and inter-alpha trypsin inhibitor heavy chain 4. Oxidized phospholipids binds the cell membrane-localized HSPA5 chaperon protein associated with its cofactor DNAJC1; this binding leads to HSPA5 trafficking to caveolin-enriched microdomains on the cell surface with activation of Src and Fyn tyrosine kinases as well as Rac1 GTPase. These processes are important for cytoskeletal organization (PMID24829380).	Cell membrane<br>Chaperon<br>Signaling<br>Cytoskeleton
ATPIF1	*ATPase inhibitor, mitochondrial.* This gene encodes a mitochondrial ATPase inhibitor. It is important for mitochondrial respiration (PMID 24685140) and maintenance of normal mitochondrial structure (PMID 33422124); it is also involved in the regulation of mitophagy (PMID 24005319).	Mitochondrial<br>Metabolism<br>Mitophagy
H6PD	*Glucose 1-dehydrogenase;6-phosphogluconolactonase.* There are two forms of glucose-6-phosphate dehydrogenase, the G and the H form that encoded by this gene. This H form shows activity also with other hexose-6-phosphates, especially galactose-6-phosphate. The	Metabolism

	protein is thus a component of the glucose-6 phosphatase metabolic pathway.	
BORCS5 (LOH12CR1)	*BLOC-1 related complex subunit 5 (Loss of heterozygosity 12 chromosomal region 1 protein).* The gene is a part in newly described fusion oncogenes (PMID 31488873, 31222839).	Carcinogenesis
SIGIRR	*Single Ig IL-1-related receptor.* This is the signaling corecetpro for IL33 (PMID 31149943) and it is also a modulator of TNF signaling through its effects on TRAF6 levels (PMID 33123603). TRAF6 is a cytoskeletal regulator (PMID 16569657) and it is also important for the regulation of NFκB signaling and activation of autophagy through interactions with Beclin-1 (PMID 29929436, 30258449).	Autophagy<br>IL33/TNF<br>Cytoskeleton<br>Intracellular signaling<br>NFκB
PGPEP1	*Pyroglutamyl-peptidase 1.* The encoded cysteine protease cleaves amino terminal pyroglutamate residues from protein substrates. It is a regulator of transcription through its effects on E2F3 (PMID 33386518); through this mechanism it can also regulate autophagy (PMID 33340658) and its mitochondrial form is also important for regulation of hypoxia-induced mitophagy (PMID 30740539).	Protease<br>Transcription<br>Mitophagy<br>Autophagy
LYST	*Lysosomal-trafficking regulator.* This encoded protein regulates intracellular protein trafficking in endosomes. Mutations in this gene are associated with Chediak-Higashi syndrome, a lysosomal storage disorder.	Lysosome<br>Endosome
CDC42SE2	*CDC42 small effector protein 2.*	
LY75	*Lymphocyte antigen 75.* This is an endocytic mannose receptor (PMID 26871602). It is important for intracellular trafficking and delivery to the endosomal compartment (PMID 17163964). The molecule has an intracellular trafficking route from clathrin-coated pits through late endosomes and lysosomes back to the cell surface.	Endosome<br>Lysosome
STK38L	Serine/threonine-protein kinase 38-like. *STK38L* encodes a serine/threonine kinase. This kinase seems to be important in hippo signaling and it seems to be a regulator of autophagy/mitophagy possibly through effects on the formation of the early phagosome (PMID 27213455).	Hippo signaling<br>Autophage<br>Mitophagy
HDHD3	*Haloacid dehalogenase-like hydrolase domain-containing protein 3.*	
DAP3	*28S ribosomal protein S29, mitochondrial.* Mammalian mitochondrial ribosomal proteins are encoded by nuclear genes and help in protein synthesis within the mitochondrion. Mitochondrial ribosomes (mitoribosomes) consist of a small 28S subunit and a large 39S subunit. This 28S subunit protein participates in apoptotic pathways which are initiated by tumor necrosis factor-α, Fas ligand, and interferon-γ. This protein potentially binds ATP/GTP and might be a functional partner of the mitoribosomal protein S27. The protein is a regulator of autophagy (PMID 26306039, 25738636).	Mitochondria<br>Apoptosis<br>Autophagy
GTF2E2	*Transcription initiation factor IIE subunit beta.* The general transcription factor IIE (TFIIE) is part of the RNA polymerase II transcription initiation complex. The protein encoded by this gene represents the beta subunit of TFIIE.	Transcription
LEPRE1	Prolyl 3-hydroxylase 1. This enzyme is a member of the collagen prolyl hydroxylase family. These enzymes are localized to the endoplasmic reticulum and their activity is required for proper collagen synthesis and assembly.	Extracellular matrix<br>Endoplasmatic reticulum
DPM3	*Dolichol-phosphate mannosyltransferase subunit 3. The encoded molecule* serves as a donor of mannosyl residues on the lumenal side of the endoplasmic reticulum (ER). Lack of Dol-P-Man results in defective surface expression of GPI-anchored proteins. Dol-P-Man is synthesized from GDP-mannose and dolichol-phosphate on the cytosolic side of the ER by the enzyme dolichyl-phosphate mannosyltransferase.	Endoplasmatic reticulum

USP3	*Ubiquitin carboxyl-terminal hydrolase 3.* This is a deubiquitinating enzyme.	Ubiquitin
YBX1	*Nuclease-sensitive element-binding protein 1.* This gene encodes a highly conserved cold shock domain protein that has broad nucleic acid binding properties. The encoded protein functions as both a DNA and RNA binding protein and has been implicated in numerous cellular processes including regulation of transcription and translation, pre-mRNA splicing, DNA reparation and mRNA packaging. This protein is also a component of messenger ribonucleoprotein (mRNP) complexes and may have a role in microRNA processing. This protein can be secreted through non-classical pathways and functions as an extracellular mitogen. The protein is a regulator of apoptosis and autophagy (PMID 32561752, 33312753)	Autophagy<br>RNA binding<br>DNA binding<br>Extracellular release
CSDE1	*Cold shock domain-containing protein E1. T*he autophagic receptor P62/SQSTM1 can form aggregates that colocalize with foci concentrating the RNA binding protein UNR/CSDE1.	Autophagy
PRMT1	*Protein arginine N-methyltransferase 1.* This gene encodes a member of the protein arginine N-methyltransferase (PRMT) family. Post-translational modification of target proteins by PRMTs plays an important regulatory role in many biological processes, whereby PRMTs methylate arginine residues. The encoded protein is a type I PRMT and is responsible for the majority of cellular arginine methylation activity. Experimental models suggest that the molecule is involved in autophagy (PMID 24140420, 26828939).	Autophagy<br>Arginine methylation
DCTD	*Deoxycytidylate deaminase. T*he encoded protein catalyzes the deamination of dCMP to dUMP, the nucleotide substrate for thymidylate synthase.	Nucleotide
HGSNAT	*Heparan-alpha-glucosaminide N-acetyltransferase.* This lysosomal acetyltransferase is one of several enzymes involved in the lysosomal degradation of heparin sulfate. Mutations in this gene are associated with Sanfilippo syndrome C, one type of the lysosomal storage disease mucopolysaccaridosis III, which results from impaired degradation of heparan sulfate. Lysosomal storage of heparan sulfate causes mitochondrial defects, altered autophagy (PMID 25998837).	Lysosome<br>Autophagy
EPB41L2	*Band 4.1-like protein 2.* This is a membrane skeletal protein (PMID 9598318).	Cell membrane
TUBA1A	*Tubulin alpha-1A chain.* Microtubules of the eukaryotic cytoskeleton perform essential and diverse functions and are composed of a heterodimer of alpha and beta tubulins. The alpha and beta tubulins represent the major components of microtubules, while gamma tubulin plays a critical role in the nucleation of microtubule assembly.	Cytoskeleton
UGDH	*UDP-glucose 6-dehydrogenase.* The protein converts UDP-glucose to UDP-glucuronate and thereby participates in the biosynthesis of glycosaminoglycans such as hyaluronan and heparan sulfate. These glycosylated compounds are common components of the extracellular matrix The expression of this gene is up-regulated by transforming growth factor beta and down-regulated by hypoxia.	Glycosylation
POLD3	*DNA polymerase delta subunit 3.* This protein is the 66-kDa subunit of DNA polymerase delta. DNA polymerase delta possesses both polymerase and 3' to 5' exonuclease activity and plays a critical role in DNA replication and repair. The encoded protein plays a role in regulating the activity of DNA polymerase delta.	DNA replication<br>DNA repair
FAF1	*FAS-associated factor 1.* Interaction of Fas ligand (TNFSF6) with the FAS antigen (TNFRSF6) mediates apoptosis. The protein encoded by this gene binds to FAS antigen and can initiate apoptosis or enhance apoptosis initiated through FAS antigen. The protein is also a regulator of the autophagy-lysosome pathway; it can also activate the mTORpathway with subsequent effects on autophagosome formation (PMID 33749937).	Apoptosis<br>Autophagy<br>mTOR
NUDCD3	*NudC domain-containing protein 3.* The protein maintains the stability of dynein intermediate chain; dynein is a family of cytoskeletal motor proteins that move along microtubules in cells. Depletion of this gene product results in aggregation and degradation of dynein intermediate chain, mislocalization of the dynein complex from kinetochores, spindle microtubules, and spindle poles, and loss of gamma-tubulin from spindle poles. The protein localizes to the Golgi apparatus during interphase, and levels of the protein increase after the G1/S transition.	Cytoskeleton<br>Golgi<br>Cell cycle

NPM3	*Nucleoplasmin-3.* The protein is related to the nuclear chaperone phosphoproteins, nucleoplasmin and nucleophosmin. It localizes primarily to the nucleus. Based on its similarity to nucleoplasmin and nucleophosmin, this protein likely functions as a molecular chaperone in the cell nucleus. It also functions as a transcriptional regulator (PMID 20073534).	Nucleus<br>Chaperon<br>Transcription
MMS19	*MMS19 nucleotide excision repair protein homolog.* The protein regulates spindle microtubule assembly (PMID 33211700), protects the mitochondrial genome from oxidative damage (PMID 29035693) and is a regulator of the cell cycle (PMID 29361561).	Mitochondria<br>Oxidative stress<br>Cell cycle
HK2	*Hexokinase-2.* Hexokinases phosphorylate glucose to produce glucose-6-phosphate, the first step in most glucose metabolism pathways. It localizes to the outer membrane of mitochondria.	Metabolism<br>Mitochondria
IPO4	*Importin-4.* The protein binds histones and import them to the nucleus (PMID 30177574).	Nuclear import
GPX7	*Glutathione peroxidase 7.* The protein is important in redox homeostasis (PMID 27186289).	Redox homeostasis
TSTD1	*Thiosulfate sulfurtransferase/rhodanese-like domain-containing protein 1.* Experimental studies suggest that the protein has a role in sulfide-based signaling.	Signaling
ZNF316	*Zinc finger protein 316.*	
CRIP2	*Cysteine-rich protein 2.* This gene encodes a putative transcription factor with two LIM zinc-binding domains.	Transcription
CALU	*Calumenin.* The product is a calcium-binding protein localized in the endoplasmic reticulum and it is involved in functions as protein folding and sorting.	Endoplasmatic reticulum<br>Protein sorting
SERPINH1	*Serpin H1.* This protein is member of the serpin superfamily of serine proteinase inhibitors. The encoded protein is localized to the endoplasmic reticulum and plays a role in collagen biosynthesis as a collagen-specific molecular chaperone.	Endoplasmatic reticulum
FAM105A	*Inactive ubiquitin thioesterase FAM105A.* The protein is a deubiquitinase (PMID 27523608). It is a regulator of initiation and maturation of autophagy through stabilization of ATG13 (PMID 32543267).	Autophagy<br>ATG13
TUBB6	Tubulin beta-6 chain. Microtubules of the eukaryotic cytoskeleton are composed of a heterodimer of alpha and beta tubulins. The alpha and beta tubulins represent the major components of microtubules, while gamma tubulin plays a critical role in the nucleation of microtubule assembly.	Cytoskeleton
OGFOD1	*Prolyl 3-hydroxylase OGFOD1.* This is a ribosomal protein (PMID 31112528).	Ribosome
DPYSL3	*Dihydropyrimidinase-related protein 3.* This is a cell adhesion protein (PMID 29514686).	Adhesion
TPPP3	*Tubulin polymerization-promoting protein family member 3.* The protein is a member of the TPPP family and can induce tubulin polymerization and microtubule bundling. It is also involved in intracellular signaling/NFκB signaling (OMID 20571904)	Cytoskeleton<br>NFκB

Table S4. A description of proteins that show significantly different levels when comparing AML cells showing strong and weak antiproliferative effects of chloroquine, a summary of the bioinformatical analyses. Cluster 1 refers to AML cells with less than 40% reduction in AML cell proliferation by chloroquine and cluster 2 refers to cells showing at least 50% inhibition.

Gene names	Cluster 1 Count	Cluster 2 Count	Cluster 1 Median	Cluster 2 Median	Cluster 2/cluster 1 Fold change	Z-score	p-value
ITGB3	8	8	19.15	23.73	4.57	0	0.030254
ITGA2B	8	8	19.80	23.75	3.96	0	0.04016
SARDH	7	4	20.84	24.25	3.40	0	0.002755
PPBP	8	8	23.26	25.92	2.66	0	0.049851
HLA-E	8	8	21.03	23.24	2.21	1.66089E-13	0.044887
SLC2A5	4	7	18.39	20.46	2.06	4.91807E-12	0.02466
SYTL1	8	8	20.89	22.48	1.59	7.4616E-08	0.041554
TTN	8	8	24.15	25.44	1.29	9.00935E-06	0.002365
SUGP2	8	8	21.40	22.64	1.24	1.78662E-05	0.040075
WDR81	8	8	21.18	21.93	0.75	0.005886163	0.003656
SAP30L	8	8	21.43	22.17	0.74	0.006223731	0.047267
DNAJC1	7	7	20.18	20.92	0.74	0.006341407	0.028401
ATPIF1	8	8	23.94	24.66	0.71	0.007794157	0.04889
H6PD	8	8	22.56	23.21	0.65	0.013138167	0.034353
LOH12CR1	6	5	20.21	20.86	0.65	0.013507151	0.036574
SIGIRR	8	7	21.02	21.66	0.64	0.014756062	0.014385
PGPEP1	3	3	21.93	22.52	0.59	0.021687326	0.037259
LYST	6	3	21.15	21.74	0.59	0.022745198	0.043091
CDC42SE2	8	8	22.22	22.77	0.55	0.030670466	0.009762
LY75	8	6	21.27	21.78	0.51	0.040218416	0.019948
STK38L	7	6	21.29	21.80	0.50	0.042355765	0.02646
HDHD3	8	8	24.84	25.34	0.50	0.043680702	0.036375
DAP3	8	8	22.84	22.28	-0.56	0.033196161	0.03349
GTF2E2	8	8	22.57	22.00	-0.57	0.031322766	0.000468
LEPRE1	8	8	22.73	22.16	-0.57	0.03042704	0.036735
DPM3	8	8	22.55	21.93	-0.61	0.022781345	0.004478
USP3	8	8	22.28	21.67	-0.61	0.022225688	0.034665
YBX1	8	8	27.28	26.66	-0.63	0.019605251	0.002811
CSDE1	8	8	23.14	22.46	-0.67	0.013514094	0.034337
PRMT1	8	8	24.82	24.14	-0.69	0.012057231	0.03876
DCTD	8	8	22.81	22.12	-0.69	0.01136674	0.048111
HGSNAT	7	5	21.32	20.62	-0.69	0.011094769	0.014334
EPB41L2	6	6	22.73	22.04	-0.70	0.011015639	0.010487
TUBA1A	8	8	25.49	24.79	-0.70	0.01013566	0.032952
UGDH	8	7	21.06	20.33	-0.73	0.007848063	0.015658
POLD3	8	8	21.82	21.07	-0.75	0.006676012	0.029528
FAF1	8	8	22.05	21.28	-0.77	0.005333	0.042206
NUDCD3	7	6	22.11	21.32	-0.80	0.00422667	0.013153
NPM3	8	8	24.61	23.75	-0.86	0.002248225	0.015499
MMS19	8	8	22.81	21.95	-0.87	0.002009659	0.043714

HK2	8	7	22.63	21.71	-0.92	0.001067627	0.047578
IPO4	8	8	22.79	21.85	-0.94	0.000873672	0.044711
GPX7	8	8	24.15	23.14	-1.01	0.000377053	0.012472
TSTD1	8	8	23.70	22.56	-1.15	6.20707E-05	0.014876
ZNF316	8	7	24.02	22.77	-1.26	1.19693E-05	0.018788
CRIP2	6	5	23.50	22.24	-1.26	1.10665E-05	0.030648
CALU	5	3	22.18	20.57	-1.61	2.94381E-08	0.037073
SERPINH1	8	8	23.96	22.09	-1.87	1.21351E-10	0.0442
FAM105A	7	7	22.07	20.06	-2.02	4.23306E-12	0.038792
TUBB6	7	7	25.02	22.75	-2.27	6.99441E-15	0.041034
OGFOD1	6	5	25.28	22.92	-2.36	0	0.00718
DPYSL3	7	6	23.55	20.98	-2.57	0	0.046475
TPPP3	7	5	24.63	20.80	-3.83	0	0.003569

Table S5. The complete list of 99 significantly increased genes (F-score >1.0 and FC value >1.0) for patient samples with high sensitivity to chloroquine. The genes are listed in alphabetical order. The table presents the gene name and the full protein name; the potential role in AML is briefly described and the corresponding references are given.

GENE ID	FULL NAME	POTENTIAL ROLE IN AML	PUBMED ID
ADA	Adenosine deaminase	High levels of adenosine deaminase are associated with unfavourable outcome in AML	7049266
AHCY	Adenosylhomocysteinase		
AK2	Adenylate kinase 2		
APRT	Adenine phosphoribosyltransferase		
ARMET/ MANF	Mesencephalic astrocyte derived neurotrophic factor		
ATP5G1	ATP synthase membrane subunit c locus 1		
C11orf1	Chromosome 11 open reading frame 1		
C5orf15	Chromosome 5 open reading frame 15		
CCDC56/ COA3	Cytochrome c oxidase assembly factor 3		
CCL23	C-C motif chemokine ligand 23	Chemokine involved in leukemogenesis	17339182
CNST	Consortin, connexin sorting protei	Connexins are expressed in AML	25529637
COPB1	COPI coat complex subunit beta 1		
CTSC	Cathepsin C		
CWC15	CWC15 spliceosome associated protein homolog		
DCTN3	Dynactin subunit 3		
DKC1	Dyskerin pseudouridine synthase 1		
DNTT	DNA nucleotidylexotransferase		
DUSP23	Dual specificity phosphatase 23		
EBNA1BP2	EBNA1 binding protein 2		
EBPL	EBP like		
FMC1	Formation of mitochondrial complex V assembly factor 1 homolog		
GAPT	GRB2 binding adaptor protein, transmembrane		
GLO1	Glyoxalase I	Decrease of glyoxalase I activity is associated with differentiation in AML	3166382
GNG10	G protein subunit gamma 10		
GNL2	G protein nucleolar 2		
GPR56	G protein-coupled receptor 56	Possible involvement in AML leukemogenesis	27063597
GSTM1	Glutathione S-transferase mu 1	Polymorphisms in GSTM1 increase the risk of developing AML	24854448
GSTM2	Glutathione S-transferase mu 2		
GUCY1A3	Guanylate cyclase 1 soluble subunit alpha 1	Associated with a leukemic stem cell gene signature in AML	21177505
HIST1H1C	H1.2 linker histone, cluster member		
HSH2D	Hematopoietic SH2 domain containing		
HSPA9	Heat shock protein family A (Hsp70) member 9	HSP70 inhibitors have antileukemic activity	23586877
HSPD1P5	Heat shock protein family D (Hsp60) member 1 pseudogene 5		
IGLL3	Immunoglobulin lambda like polypeptide 3, pseudogene		
ITGAE	Integrin subunit alpha E		
ITGB1BP1	Integrin subunit beta 1 binding protein 1		
LAT2	Linker for activation of T cells family member 2	Possibly involved in the t(8;21)((q22;q22)) translocation in acute leukemia	21488857

LPIN1	Lipin 1		
LYRM1	LYR motif containing 1		
LYSMD2	LysM domain containing 2		
MAGED1	MAGE family member D1	An AML-associated antigen	25092142
MAP4K1	Mitogen-activated protein kinase kinase kinase kinase 1	High expression of MAP4K1 has been associated to poor prognosis in AML	31522654
MIR155HG	MIR155 host gene	miR-155 has a potential negative prognostic impact in AML	23650424
MRPL21	Mitochondrial ribosomal protein L21		
MRPL24	Mitochondrial ribosomal protein L24		
MRPL36	Mitochondrial ribosomal protein L36		
MRPL45	Mitochondrial ribosomal protein L45		
MRPL54	Mitochondrial ribosomal protein L54		
NDUFA13	NADH:ubiquinone oxidoreductase subunit A13		
NDUFA8	NADH:ubiquinone oxidoreductase subunit A8		
NDUFB6	NADH:ubiquinone oxidoreductase subunit B6		
NENF	Neudesin neurotrophic factor		
NME4	NME/NM23 nucleoside diphosphate kinase 4		
NT5C3	5'-nucleotidase, cytosolic IIIA	NT5C3 polymorphism associated with response to chemotherapy in AML	25000516
NUDT1	Nudix hydrolase 1		
NUDT5	Nudix hydrolase 5		
PAICS	Phosphoribosylaminoimidazole carboxylase and Phosphoribosylaminoimidazole-succinocarboxamide synthase		
PARP1	Poly(ADP-ribose) polymerase 1	High PARP-1 expression is a predictor for poor survival in AML patients	30472087
PBX3	PBX homeobox 3	Found to interact in MLL-rearranged AML	26747896
PDLIM1	PDZ and LIM domain 1		
PFKP	Phosphofructokinase, platelet		
PHB2	Prohibitin 2		
PIM1	Pim-1 proto-oncogene, serine/threonine kinase	Expressed in AML patients and possible prognostic marker	28851457
PIN1	Peptidylprolyl cis/trans isomerase, NIMA-interacting 1	Increased expression in AML, and a potential pharmacological target	29848341
POLR3GL	RNA polymerase III subunit G like		
PRMT1	Protein arginine methyltransferase 1	Promotes survival and growth of FLT3-ITD^{+} AML cells	3121718
PROK2	Prokineticin 2		
PSMD4	Proteasome 26S subunit, non-ATPase 4		
RAB37	RAB37, member RAS oncogene family		
RPA3	Replication protein A3		
RPL12P6	Ribosomal protein L12 pseudogene 6		
RPS26P2	Ribosomal protein S26 pseudogene 2		
RPS26P53	RPS26P53 ribosomal protein S26 pseudogene 53		
RPS26P55	Ribosomal protein S26 pseudogene 55		
SELS			
SLC2A5	Solute carrier family 2 member 5	Enhanced fructose utilization in AML cells, and a potential therapeutic target	27746145
SLC39A3	Solute carrier family 39 member 3		
SLC9A3R1	SLC9A3 regulator 1		
SMC4	Structural maintenance of chromosomes 4	Involved in leukemogenesis related to chromosomes condensing	29043883
SOCS2	Suppressor of cytokine signaling 2	High expression of SOCS2 associated with	24559289

		negative outcome in AML	
SPOP	Speckle type BTB/POZ protein		
SSB	Small RNA binding exonuclease protection factor		
SUCLG1	Succinate-CoA ligase GDP/ADP-forming subunit alpha	Associated with possible prognostic gene profile in AML	29138577
TBCB	Tubulin folding cofactor B		
TECR	Trans-2,3-enoyl-CoA reductase		
TMED9	Transmembrane p24 trafficking protein 9		
TOMM22	Translocase of outer mitochondrial membrane 22		
TSEN34	tRNA splicing endonuclease subunit 34		
UCKL1	Uridine-cytidine kinase 1 like 1		
UQCRC1	Ubiquinol-cytochrome c reductase core protein 1		
WDR61	WD repeat domain 61		
ZMYM6	Zinc finger MYM-type containing 6		
ZNF207	Zinc finger protein 207		

Table S6. The complete list of 74 significantly increased genes (F-score >1.0 and FC value >1.0) in patient samples with upregulated release of soluble mediators after chloroquine treatment. The genes are listed in alphabetical order. The table presents the gene name and the full protein name; the potential role in AML is briefly described and the corresponding references are given.

GENE ID	FULL NAME	POTENTIAL ROLE IN AML	PUBMED ID
ADAP1/ CENTA1	Arf GAP with dual PH domains 1		
ANXA2P1	Annexin A2 pseudogene 1		
ANXA5	Annexin A5		
ARL4A	ADP ribosylation factor like GTPase 4A		
BLOC1S1	Biogenesis of lysosomal organelles complex 1 subunit 1		
BLOC1S1	Biogenesis of lysosomal organelles complex 1 subunit 1		
C5orf15	Chromosome 5 open reading frame 15		
CCL23	C-C motif chemokine ligand 23	Chemokine involved in leukemogenesis	
CDKN1A	Cyclin dependent kinase inhibitor 1A		
CKS1B	CDC28 protein kinase regulatory subunit 1B		
CNIH4	Cornichon family AMPA receptor auxiliary protein 4		
COMMD8	COMM domain containing 8		
CRLS1	Cardiolipin synthase 1		
CTSH	Cathepsin H		
DHRS9	Dehydrogenase/reductase 9		
DPM1	Dolichyl-phosphate mannosyltransferase subunit 1		
DYNLT1	Dynein light chain Tctex-type 1	Possible involved in the t(7;11)(p15;p15) translocation in acute leukemia	12446457
EAF2	ELL associated factor 2	Possible involved in the (11;19)(q23;p13.1) translocation in acute leukemia	12446457
EIF5A	Eukaryotic translation initiation factor 5A-2	Involved in chemoresistance in AML	30745844
EVI2A	Ecotropic viral integration site 2A		
FABP5	Fatty acid binding protein 5		
FAM45A	Family with sequence similarity 45		
FGL2	Fibrinogen like 2		
FLT3	Fms related tyrosine kinase 3	Both FLT3 mutation and FLT3 overexpression have prognostic impact in AML	
FUT4	Fucosyltransferase 4		
GLB1	Galactosidase beta 1		
GRN	Granulin precursor		
GTF2H5	General transcription factor IIH subunit 5		
HEBP2	Heme binding protein 2		
HLA-DQA1	Major histocompatibility complex, class II, DQ alpha 1		
HLA-DRB5	Major histocompatibility complex, class II, DR beta 5		
HMGB1P37	High mobility group box 1 pseudogene 37		
IAH1	Isoamyl acetate-hydrolyzing esterase		
IGFBP7	Insulin like growth factor binding protein 7	Induces differentiation and loss of survival in LSCs	30540936
IRF8	Interferon regulatory factor 8	Possible tumor-suppressor role for IRF8 in AML	30266821
ITGAE	Integrin subunit alpha E		
KYNU	Kynureninase	Found downregulated in IDH mutated	21647152

		AML cases	
LSM3P4	U6 small nuclear RNA and mRNA degradation associated pseudogene 4		
LY86	Lymphocyte antigen 86	Identified in gene signature for LSCs, associated with chemoresistance	20371479
LY96	Lymphocyte antigen 96		
MAPBPIP	Late endosomal/lysosomal adaptor, MAPK and MTOR activator 2		
MCUB/ CCDC109B	Mitochondrial calcium uniporter dominant negative subunit beta		
MMADHC	Metabolism of cobalamin associated D		
MNDA	Myeloid cell nuclear differentiation antigen	Correlated with myeloid and monocytic differentiation in acute leukemia	2259228
MRPL13	Mitochondrial ribosomal protein L13		
MRPL36	Mitochondrial 54S ribosomal protein YmL36		
MS4A7	Membrane spanning 4-domains A7		
NDUFA13	NADH ubiquinone oxidoreductase subunit A13		
NDUFB9	NADH: ubiquinone oxidoreductase subunit B9		
NUDT5	Nudix hydrolase 5		
PECAM1	Platelet and endothelial cell adhesion molecule 1	Bone marrow retention of AML cells depends on PECAM1 expression	17875702
PFKP	Phosphofructokinase, platelet		
PGAM1	Phosphoglycerate mutase 1		
PPP2R3C	Protein phosphatase 2 regulatory subunit B"gamma		
PQLC3	Solute carrier family 66 member 3		
PSMB8	Proteasome 20S subunit beta 8	Immunoproteasome subunits critical for malignant cells to escape immune recognition	23770850
RAB11 FIP1	RAB11 family interacting protein 1		
RPL21P14	Ribosomal protein L21 pseudogene 14		
SCPEP1	Serine carboxypeptidase 1		
SERPINB2	Serpin family B member 2	Important for stem cells in response to various existing chemicals	29925837
SLC35A1	Solute carrier family 35 member A1		
SLC7A7	Solute carrier family 7 member 7		
SMYD3	SET and MYND domain containing 3		
SNHG8	Small nucleolar RNA host gene 8		
SNX2	Sorting nexin 2	Found to interact in MLL-rearranged AML	11438682
TRAPPC5	Trafficking protein particle complex 5		
UCP2	Uncoupling protein 2		
UQCRC1	Ubiquinol-cytochrome c reductase core protein 1		
UTP6	UTP6 small subunit processome component		
VCAN	Versican	Possible prognostic biomarker in AML	29861382
VPS28	VPS28 subunit of ESCRT-I		
WDR61	WD repeat domain 61		
ZFR	Zinc finger RNA binding protein		

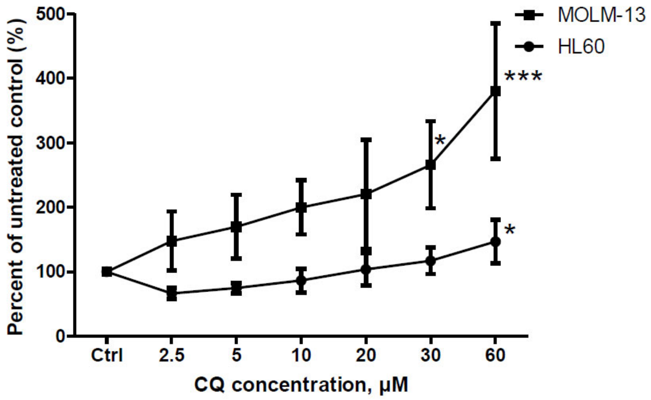

Figure S1. The inhibitory effect of chloroquine (CQ) on autophagy in AML cell lines. The Cyto-ID® autophagy detection kit for flow cytometry was used to monitor autophagy in HL60 and MOLM-13 cells after treatment with or without different concentrations of CQ for 18 hours (2.5-60 µM). Autophagy was measured as fluorescence (incorporation of CytoID reagent into autophagic vesicles) and results are presented on the y-axis as percentage of fluorescence of untreated controls (Ctrl). CQ leads to accumulation of autophagic vesicles in a dose-dependent manner. MOLM-13 seems to have a higher autophagic flux than HL60 cells. The results are presented as the mean values (± standard deviation) of three independent experiments (* $p<0.05$, *** $p<0.0001$; one-way ANOVA with Dunnett's multiple comparison post test).

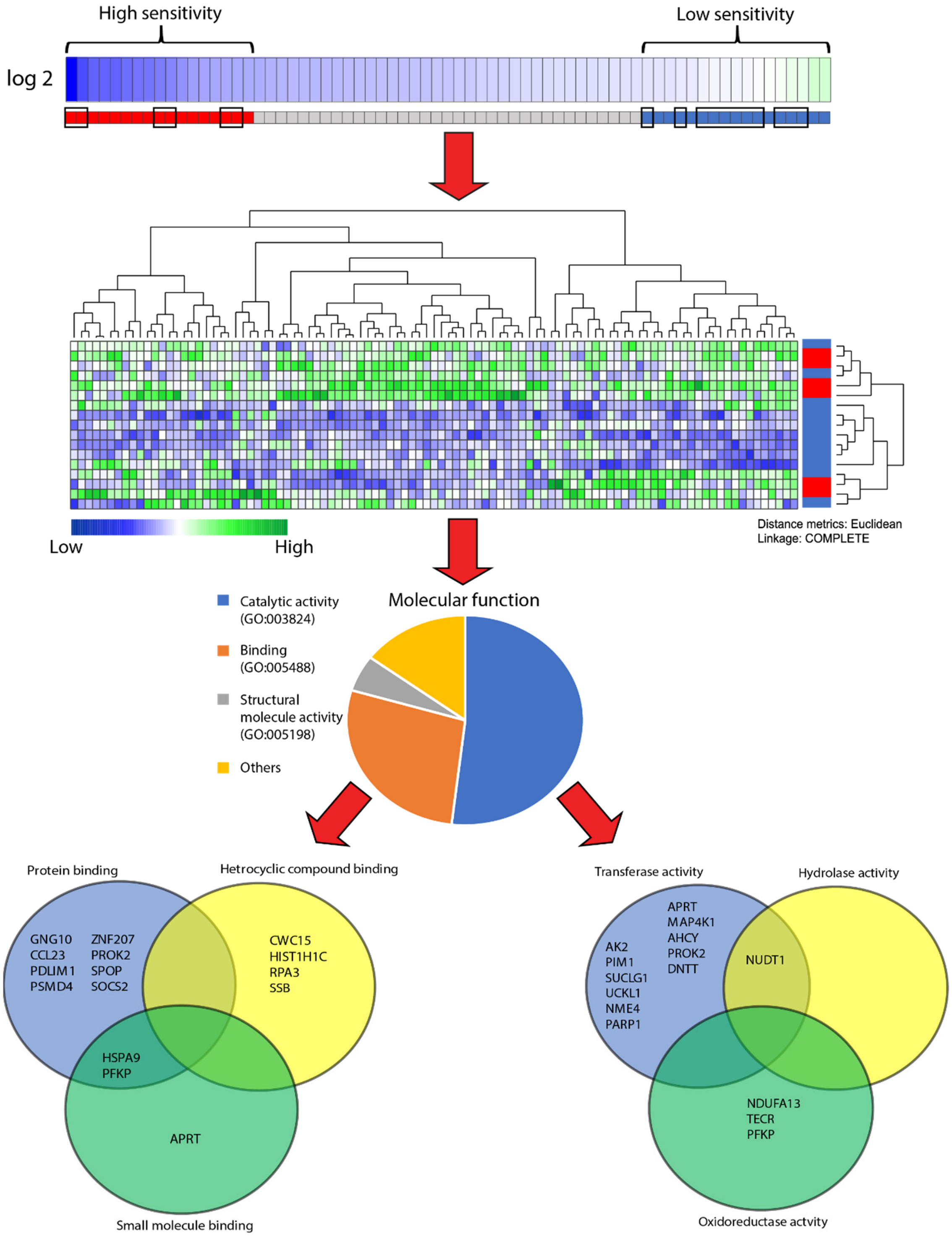

Figure S2. Profiling of gene expression data based on antiproliferative effects of chloroquine on primary AML cells. (UPPER) For 69 AML patients, the proliferation of AML cells after treatment with 2.5 µM chloroquine was compared to the proliferation of untreated controls. Proliferation was analyzed using the [^{3}H]-thymidine incorporation assay, and we used the log2 ratio of the proliferation values to create a heat map (illustrated in upper part of figure). Based on this heat map, the patient cohort was further divided into quartiles, where the quartile at the far left side of the heat map (shown in red) included patients that had the most decreased proliferation after chloroquine treatment (high sensitivity to chloroquine), while the quartile to the far right (shown in blue) had unaltered or high relative proliferation after chloroquine treatment (low sensitivity to chloroquine). (MIDDLE) Gene expression data for six patients found in the quartile with high sensitivity to

chloroquine was then compared with 11 patients found in the quartile with low sensitivity to chloroquine (patients marked in boxes). Based on ANOVA (F-score >1.0 and FC value >1.0), we identified 99 genes upregulated among patients that were highly sensitive to chloroquine (a strong antiproliferative effect), illustrated by the unsupervised hierarchical cluster analysis shown in the middle part of the figure. (LOWER) The genes encoding proteins with a known function were classified according to the PANTHER database, and by using the term molecular function we identified the subcategories "Catalytic activity" and "Binding" as the two most prominent terms among the upregulated genes. Furthermore, we identified the three largest subterms belonging to these subcategories, and the figure shows the genes upregulated in each subterm (see colored circles at bottom of figure). All 99 identified upregulated genes are listed in Table S5.

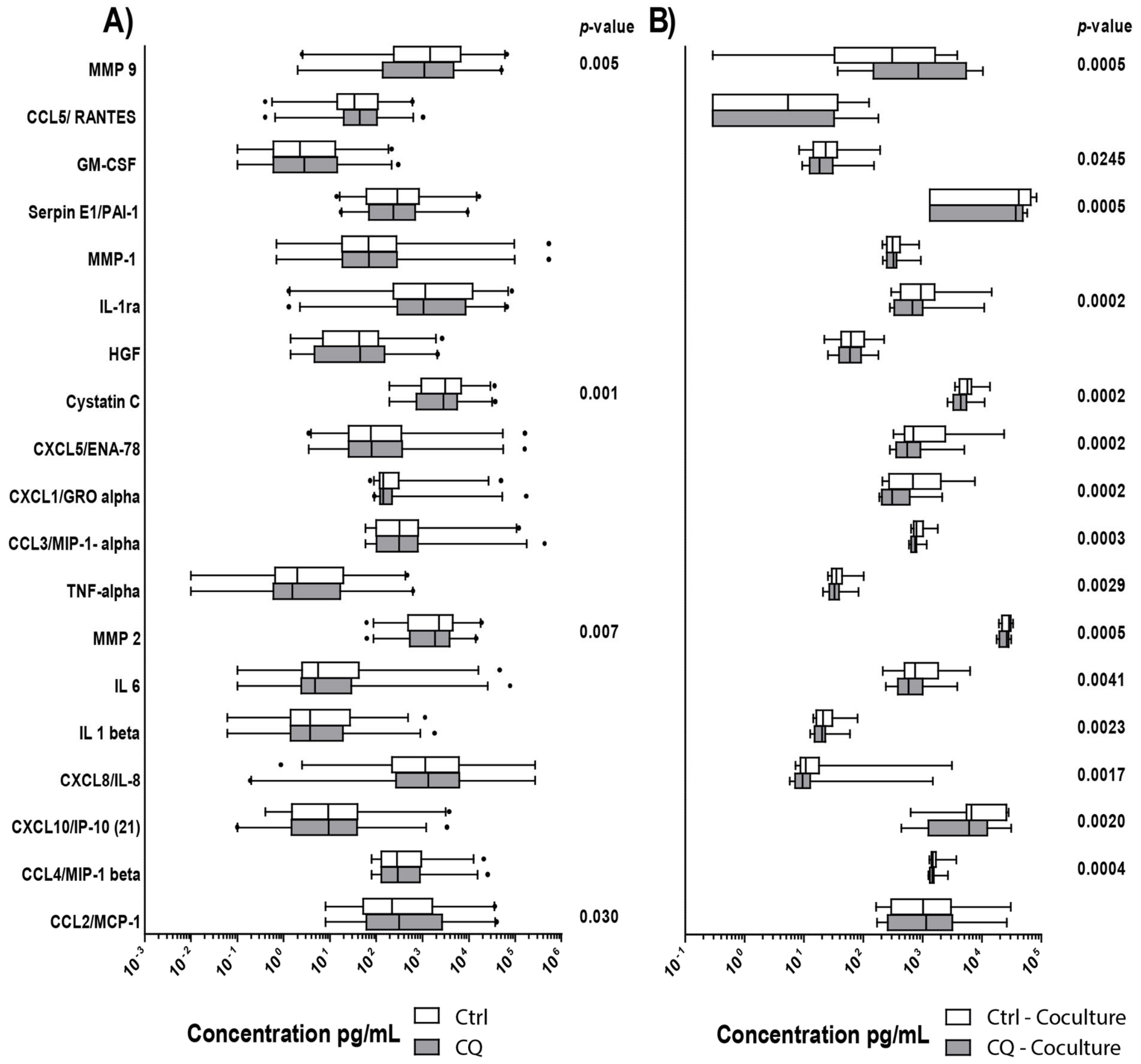

Figure S3. Effect of chloroquine treatment on the constitutive release of soluble mediators in AML cell cultures and in AML-MSC cocultures. A) Primary AML cells from 72 patients were incubated for 48 hours with CQ 5 µM or medium alone before supernatants were harvested and the concentrations of the soluble mediators were determined. The results are presented as the median levels, 25/75 percentiles (box), 5/95 percentiles (whiskers) and outliers, for CQ treated cultures (CQ) and untreated cultures (Ctrl). Significant alterations are shown in the figure (p-value <0.05, Wilcoxon signed rank test). **B)** Primary AML cells derived from 14 patients were cocultured with normal MSCs derived from one donor. The cells were cultured together in transwell cocultures, i.e. the two cell types have no direct contact. After 48 hours' incubation with CQ 5 µM or in medium alone, supernatants were harvested and the concentrations of the soluble mediators were determined. The results are presented as the median levels, 25/75 percentiles (box), 5/95 percentiles (whiskers) and outliers, for CQ treated cocultures (CQ - Coculture) and untreated cocultures (Ctrl - Coculture). Significant alterations (p-value <0.05) are shown in the figure (Wilcoxon signed rank test).

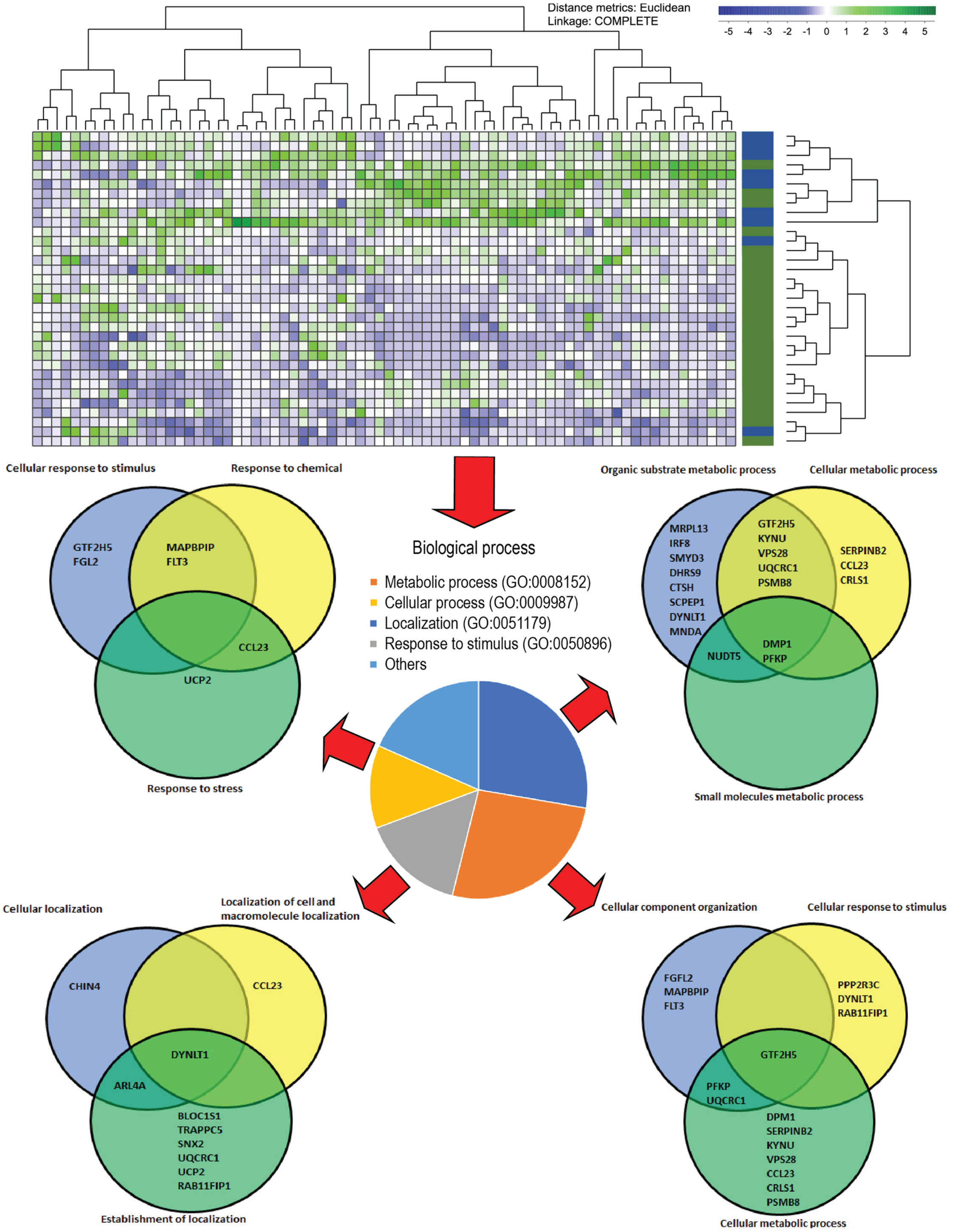

Figure S4. Gene expression profiles associated with levels of soluble mediators released by AML cells after chloroquine treatment. Total gene expression profiling was available for 33 AML patients included in the study, and we compared the 24 patients with low/intermediate mediator release after chloroquine-treatment (Figure 5, the two lower subclusters) with the nine patients showing high mediator release (Figure 5, top subcluster). Using ANOVA, and by setting an F-score >1.0 and a fold change (FC) value >1.0, we identified 74 genes differently expressed between the two patient populations. These genes were used to perform an unsupervised hierarchical cluster analysis (Euclidean distance matrix, with complete linkage), which could discriminate the two patient's subsets (Upper part). Patients with high release of soluble mediators after

chloroquine-treatment are shown in blue, while patients with low/intermediate release are shown in green. The genes encoding proteins with a known function were classified using the PANTHER database. By using the term biological process, we identified metabolic process, cellular process, localization and response to stimulus as the four most frequent subgroups. Finally, we searched for GO-terms in these four subgroups and the single genes for each of these subgroups are shown in the colored circles (lower part).
